# Recent Advances in Functionalized Mesoporous Silica Frameworks for Efficient Desulfurization of Fuels

**DOI:** 10.3390/nano10061116

**Published:** 2020-06-05

**Authors:** Shruti Mendiratta, Ahmed Atef Ahmed Ali

**Affiliations:** Department of Chemical and Petroleum Engineering, University of Calgary, Calgary, AB T2N1N4, Canada

**Keywords:** mesoporous silica, nanomaterials, desulfurization, fuel, JP-8

## Abstract

Considerable health and climate benefits arising from the use of low-sulfur fuels has propelled the research on desulfurization of fossil fuels. Ideal fuels are urgently needed and are expected to be ultra-low in sulfur (10–15 ppm), with no greater than 50 ppm sulfur content. Although several sulfur removal techniques are available in refineries and petrochemical units, their high operational costs, complex operational needs, low efficiencies, and higher environmental risks render them unviable and challenging to implement. In recent years, mesoporous silica-based materials have emerged as promising desulfurizing agents, owing to their high porosity, high surface area, and easier functionalization compared to conventional materials. In this review, we report on recent progress in the synthesis and chemistry of new functionalized mesoporous silica materials aiming to lower the sulfur content of fuels. Additionally, we discuss the role of special active sites in these sorbent materials and investigate the formulations capable of encapsulating and trapping the sulfur-based molecules, which are challenging to remove due to their complexity, for example the species present in JP-8 jet fuels.

## 1. Introduction

According to the World Health Organization (WHO), exposure to air pollution is the topmost environmental health risk factor, having led to one in eight global deaths in 2016 [1]. Reducing the greenhouse gas emissions is listed as one of the sustainable goals of the United Nations which directed their climate change mitigation efforts to apply stringent regulations, promote intelligent transport systems and innovative construction techniques, and invest in cleaner energy from fossil fuels through greenhouse gas capture, storage, and sequestration [2]. As per the 2016 Climate and Clean Air Coalition (CCAC) report, fine particles (PM_2.5_) present in the air, arising as a consequence of high sulfur content in fuels, are among the major contributors to air pollution. The presence of sulfur in fuels is linked directly to increased air pollution through direct emissions of harmful sulfur oxide (SO_x_) compounds in the atmosphere, resulting in acid rain, as well as indirectly by deactivating/poisoning the catalysts used in the refining systems and decreasing the efficiencies of emission control devices [3]. For example, black carbon released from the combustion of diesel can be captured through filters; however, these filters perform well only in the presence of low sulfur concentrations. Therefore, desulfurization of fuels becomes imperative for the preservation of environment, protection of health, and for increasing the lifetime of components playing essential roles in power generation systems.

**Scheme 1:**
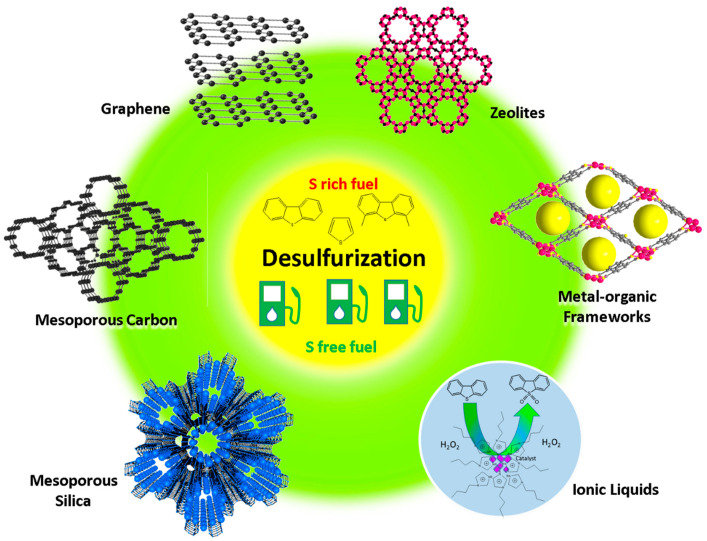
Various porous materials reported for desulfurization of fuels.

High chemical affinity of sulfur atoms for metallic cations have made metal-oxide loaded solid sorbents to play vital role in removing sulfur content at mid, as well as high, temperatures. Hydrodesulfurization (HDS) is one of the conventional methods to desulfurize fuel in refineries where organic sulfur is converted to H_2_S employing transition metal catalysts loaded on alumina substrates. Low cost transition metal oxides of Mn, Zn, Fe, Co, and Mo act as active components of these catalysts due to their high thermal stability [4,5,6,7,8,9]. However, energy demanding operating conditions involving high temperatures and pressures, low octane/cetane values, and selectivity towards simple refractory sulfur compounds in comparison to more complex aromatic species makes this process less efficient and makes room for new alternatives. Although few companies, like Akzo Nobel, ExxonMobil, and Nippon Ketjen, have come forward with a new trimetallic HDS catalyst (Mo-W-Ni, NEBULA) capable of producing diesel oil with a sulfur content as low as 10 ppm sulfur content, this process is still very energy intensive and requires hydrogen. Much effort is now being directed towards low temperature purification systems with high adsorption capacities for sulfur by exploring the efficiency of adsorbents, such as zeolites, metal–organic frameworks (MOFs), carbonaceous materials, ionic liquids (IL), and silica (Scheme 1). However, the efficiency of conventional adsorptive desulfurization methods can only be enhanced in the presence of better adsorbents or by using a combination of sulfur removal platforms.

The discovery of mesoporous molecular sieves (MCM-41) possessing hexagonal porous channels was a hallmark in materials science, not only in terms of opening avenues of new materials, such as mesoporous silica nanoparticles, mesoporous carbon nanosheets, and MOFs, for catalysis, gas adsorption, and drug delivery but also towards creation of new zeolites and their derivatives [10,11,12,13,14,15,16,17,18,19,20]. Structural marvels of mesoporous silica (MS) have also motivated researchers in recent years to investigate their performance in desulfurization of fuels, and they are being investigated as supports for metal oxides, owing to their high porosities, large surface areas, structural flexibility, and easy functionalization. High pore volumes and confined pore characteristics of one dimensional MCM-41 (with a 2–4 nm pore diameter) and two dimensional Santa Barbara Amorphous (SBA)-15 (with a 4.5–30 nm pore diameter) and their derivatives make them ideal representatives for adsorbing common aromatic S-based molecules present in fuels, such as benzothiophene (BT), dibenzothiophene (DBT), or 4,6-dimethyldibenzothiophene (4,6-DMDBT). To the best of our knowledge, this review article is the first to focus on the design and performance of mesoporous silica frameworks and the corresponding nanoparticles for desulfurization of fuels.

In this review, we discuss the synthesis, functionalization, and desulfurization performance of various MS frameworks. We focus our attention on the promising adsorption techniques using MS frameworks containing photocatalytic, redox, magnetic, ionic liquid, and carbonaceous active sites. A brief account of the synthetic strategies for preparing a MS catalytic host framework is given, along with commercial, as well as developing, desulfurization strategies. In addition, we discuss the mechanisms for removal of sulfur rich species wherever possible and make efforts to understand the role of special active sites in such sorbents. Finally, we investigate the formulations capable of encapsulating and trapping the sulfur-based molecules that are more complex and difficult to remove, such as the ones present in JP-8 and JP-5 jet fuels. In our opinion, this review will be beneficial for a wide audience working in the fields of energy production and environmental remediation.

## 2. Strategies to Synthesize Mesoporous Silica Catalytic Host Materials

High surface area of MS makes it a very promising material for the adsorptive desulfurization and hydrothermal methods. The meso-templates method is one of the conventional methods for MS catalyst preparation. A typical MS synthesis involves the self-organization of surfactant species, followed by their treatment with silica precursors. Condensation of the precursors around surfactants and their consecutive removal via reflux or calcination generates perfect MS frameworks. However, the hydrothermal method using various cationic, anionic, and neutral surfactants makes this process expensive and non-ecofriendly. Fortunately, these shortcomings can be overcome by incorporating new green and economical synthetic strategies using renewable templates. In recent years, sustainable development methods involve new templating strategies using nanocellululose, triblock copolymers, and task-specific ionic liquids as templates, instead of conventional surfactants. Some methods mentioned in this section even bypass the traditional templating strategies by using molecular imprinting techniques and single or dual phase hydrolytic conditions. 

### 2.1. Templating Strategies

#### 2.1.1. Nanocellulose Template

One of the most abundant biomaterial present in nature is cellulose, which is a low cost, reusable, and biodegradable material. Upon acid hydrolysis, this material can be transformed to form nanocellulose fibers with lengths ranging from 50 nm to a micron [21]. Nanocellulose has the potential to become an environmentally benign templating alternative. It can offer benefits of both soft and hard templates, owing to its unique structural and chemical features, including high aspect ratio and low density, as well as presence of hydrophilic surface hydroxyl groups [22,23]. Recently, nanocellulose was used as a template by Shen et al. for the development of tungsten-impregnated mesoporous silica catalysts [24]. The resulting disordered worm-like mesoporous catalysts had highly dispersed tungsten species and high surface areas in the range of 344–535 m^2^/g. It was observed that the pore diameter (2.3 nm to 10 nm) and pore volume (0.32 cm^3^/g to 0.95 cm^3^/g) increased significantly when the dosage of nanocellulose templates was increased in the precursor solution. 

#### 2.1.2. Triblock Copolymer (P123) as a Template

Several types of triblock copolymers are used in the synthesis of porous materials as the pore-directing agents. One widely used symmetric triblock copolymer with favorable characteristics is Pluronic P123, which comprises poly(ethylene oxide)–poly(propylene oxide)–poly(ethylene oxide) repeating units. P123 exhibits high hydrothermal stability, as well as amphiphilic properties, that can form stable micelles [25]. Generally, P123 can be used to form 2-D hexagonal mesoporous silica structures using acidic conditions and room temperature [26]. However, alteration of the synthesis conditions, such as the addition of sodium dodecyl sulfate (SDS) [27], n-butanol [28], or NaI [29], can result in the formation of cubic *Ia*3*d* mesoporous silica structures. In 2020, Tagaya et. al. successfully synthesized novel biocompatible slit-shaped mesoporous silica/hydroxyapatite hybrid particles using P123 and Cetyl trimethylammonium bromide (CTAB) for biological applications. [30]. It was found that the use of P123 as a template was beneficial in increasing the pore size of the mesoporous structure of synthesized particles. Moreover, the use of P123 imparted dispersion stability for the particles at the monodisperse state.

#### 2.1.3. Task-Specific Ionic Liquid Strategy

Task-specific ionic liquids (TSIL) have gained significant attention in recent years for their role in synthesizing nanoparticles and chiral moieties with tailorable characteristics (physical, chemical, and biological) [31]. A series of mesoporous silicas (which were further loaded with zirconium derived nanoparticles) was successfully synthesized from hydrolysis of the precursor material tetraethoxysilane (TEOS) in acidic medium in the presence of the ionic liquid 1-hexadecyl-3-methylimdazolium bromide, which acted as a template material for the mesoporous silicas. The synthesized mesoporous silica structures showed high capacity to be impregnated with the zirconium derived nanoparticles [32]. Similarly, high-surface-area mesoporous silicas were fabricated using the task-specific ionic liquid 1-alkyl-3-methylimidazolium hydrogen sulfate as the template material from the precursor material TEOS [33]. In addition, it was reported that the use of the long-chain ionic liquid 1-hexadecyl-3-methylimidazolium chloride can produce two-dimensional hexagonal p6mm and cubic *Ia*3*d* mesoporous silica structures, depending on the synthesis conditions from the precursor material TEOS [34].

#### 2.1.4. Polystyrene Colloidal Crystal Template

The use of colloidal crystals as templates for the synthesis of various mesoporous structures has gained adequate recognition due to their ordered interparticle pores and other desirable characteristics [35]. Yang et al. prepared bimodal ordered porous silica structures with mesoporous macropore walls, which were formed from polystyrene colloidal crystal templates. They used the seed emulsion polymerization method to prepare the polystyrene template films, which were used to prepare the silica structures from the precursor material TEOS [36]. Míguez and coworkers reported the successful fabrication of bimodal mesoporous silica structures within polystyrene colloidal crystal films that were formed on the surface of glass substrate from the precursor material TEOS using the spin-coating method [37]. In 2019, Peng et al. could achieve the synthesis of a hybrid nanostructure composed of a core of hollow mesoporous silica nanoparticles coated with a shell of pH-responsive and thermo-sensitive moieties as a model of drug delivery platform [38]. The authors used the soap-free emulsion polymerization method to prepare monodisperse polystyrene microspheres as a template for the fabrication of their hollow mesoporous silica nanoparticles, which were monodispersed with adjustable mesoporous silica shell thickness and particle size (depending on the fabrication parameters). The synthesis of ordered macroporous silica particles from the precursor material tetramethyl orthosilicate (TMOS) using polystyrene colloidal crystal as a template was also reported [39].

### 2.2. Molecular Imprinting Polymer Technology

Molecular imprinting technique offers an alternative method for deep desulfurization, where refractory organosulfur species can be easily removed under mild conditions [40]. In this technique, functionalized monomers are made to react and crosslink with template molecules. After the reaction, the template molecules are extracted leaving behind well-defined arrangement of ligands structures and tailor-made binding pockets. Compared to other structurally similar compounds, surface molecularly imprinted polymers have distinct affinity towards their templates. There are several benefits associated with their usage, such as high guest absorption and access, reduced mass transfer resistance, feasible extraction of template molecules, rapid adsorption kinetics, structural tenability, and easy synthetic protocols [41,42,43]. As binding capacity of these polymers critically depends on surface area of templates, new materials with high porosity and surface areas are in demand and mesoporous silica materials are ideal candidates for this role, thanks to their high surface areas, uniform pores, and structural and chemical tunability, as well as diverse morphologies, which makes them suitable materials for molecular imprinting technology.

### 2.3. 1D Silica Fibers and Nanowires

Development of carbon nanotube mimicking one dimensional mesoporous silica micro and nanostructures have gained much attention in recent years for applications, such as waveguides and laser components, and several different strategies are being implemented to make these materials with a diameter less than 50 nm. Electrospinning methods can generate silica fibers with an average diameter of 40 um [26], while anodic aluminium membrane strategy [27] can help in growing fibers that have small diameters of about 250 nm. In contrast, two-phase solution methods have shown the growth of mesoporous silica fibers with average diameters in the range of 1–15 um [28], and this method can even be extended to one-phase (aqueous) hydrolysis method in the presence or absence of acidic environment to produce 1D fibrous silica-based materials with 50–300 nm diameters [29]. Transforming 1D silica fibers into materials with unique pore architecture is of prime importance for materials scientists. Judicious design of mesoporous silica materials with hierarchical pores is associated with benefits, such as efficient host guest interactions, high catalytic performance, and better mass transport properties [30]. Although high porosity and large surface areas are recognized as key features for their implementation, orientation of mesopores should also be given equal importance. Studies have shown that mesopores that are parallel with the length of 1D silica fibers can limit catalyst loading and decrease active catalytic sites by blocking the longitudinal channels, whereas mesopores aligned perpendicular to the axial direction of 1D channels permit easy catalyst access and higher effective surface area [31,32].

## 3. Strategies to Desulfurize Fuel

### 3.1. Hydrodesulfurization (HDS)

Hydrodesulfurization is one of the conventional methods to desulfurize crude oil where concentration of the sulfur containing compounds is reduced. However, HDS takes place in the presence of harsh operating conditions requiring elevated temperatures in the range of 300–450 °C, hydrogen, catalysts, and high pressures ranging from 3–5 MPa, making it an expensive desulfurization technique [44]. HDS is certainly effective in removing simple thiols, thioethers, and disulfides; however, it is not successful in removing other complex heterocyclic molecules, such as dibenzothiophene (DBT), 4,6-dimethyldibenzothiophene (4,6-DMDBT), and their derivatives [44]. A typical HDS catalysis generates H_2_S gas which is converted to elemental sulfur through a Claus process [45]. Harsh catalytic conditions cause hydrogenation of olefins, followed by a negative octane rating and increased H_2_ consumption, whereas a mild HDS environment generates recombinant mercaptans through the reaction of H_2_S with olefins and are responsible for retained sulfur in final fuel. Desulfurization mechanism of DBT using hydrogen at 300 °C and 102 atm is suggested to proceed via two major pathways, direct desulfurization (DDS) and hydro desulfurization (HYD) pathways. The DDS pathway involves elimination of sulfur without hindering the aromatic rings, while HYD proceeds via selective hydrogenation of DBT aromatic rings, followed by desulfurization [44]. Molybdenum-, nickel-, cobalt-, and tungsten-based catalysts are commonly used and incorporated in the HDS process to reduce sulfur content in the form of disulfides, thioethers, and mercaptans. Performance of HDS process is greatly influenced by the choice of catalyst, and the efficiency has been found to be high in case of second and third row elements in the periodic table, owing to their unique electronic and structural characteristics. Several bimetallic blended catalysts, including alumina supported NiMo, NiW, CoW, CoMo, NiMo, and PtPd, have shown better catalytic activity in the past through highly selective and efficient hydrogenation [44,46,47,48]. In particular, nano-sized noble metals catalysts, including Pd, Pt, and Rh, have shown superior hydrogenation performance and high activities for deep hydrodesulfurization. However, their high susceptibility to sulfur poisoning due to the adsorption of H_2_S on noble metals restricts their widespread usage [49,50]. Additional drawbacks of using noble metals in hydroprocessing catalysts have been summarized by Marafi and Furimsky that include deactivation by coke deposition, inhibition by sulfur and oxygenates, poisoning by nitrogen and chlorides, deposition of contaminant metals, agglomeration of active metals, and leaching of active metals [51]. Different modes of deactivation may take place simultaneously, and adverse efforts are made for catalyst recovery by regeneration. The issue of sulfur poisoning is overcome by using phosphides of noble metals (Rh_2_P, Ru_2_P, Pd_5_P_2_), by using transition metal phosphides (Co_2_P, CoP,Ni_2_P, MoP, WP), or by using acidic supports (Al-SBA-15) [49,50]. Among various supports, SBA-15 has been used widely as a hydrodesulfurization support. Bimetallic catalysts impregnated SBA-15 (CoMo-SBA-15, NiMo-SBA-15) have shown better thiophene conversion compared to γ-Al_2_O_3_ supported catalysts [52,53]. Some excellent reports and reviews are available on this topic and reveal that mesoporous silica frameworks, including SBA-15, MCM-41, MCM-48, and KIT-6, are promising HDS agents when they are incorporated with active species, such as WS_2_, NiW, Ni_2-x_M_x_P (M = Co, Fe), MoS_2_, and NiMoO_4_, or with heteropolyacids, like H_3_PW_12_O_40_, H_3_PMo_12_O_40_, and (NH_4_)_6_Mo_7_O_24_.4H_2_O [50,54,55,56]. Cost associated with HDS and stringent fuel specifications in recent times have motivated researchers to explore new innovative strategies.

### 3.2. Oxidative Desulfurization (ODS)

ODS has emerged as a promising desulfurization technology, owing to its mild operating conditions requiring ambient temperatures and atmospheric pressures, no H_2_ prerequisites, and high efficiency towards the refractory thiophenic sulfides (DBTs). In a typical ODS process, oxidation of heavy sulfides takes place by incorporation of oxygen atoms at sulfur sites, without rupturing C–S bonds in the presence of judiciously selected oxidants, such as H_2_O_2_, O_3_, NO_2_, peroxy salts, or tertbutyl-H_2_O_2_. Out of these, H_2_O_2_ is the most widely used oxidant; however, in recent years, transition metal-based oxidizing agents (Re, Mo/W oxides, tungstophosphoric acid, MPcS and persulfates, tetra-amido macrocyclic ligands) have become popular and have been investigated in combination with hydrogen peroxide [57,58,59,60]. The success of an ODS process depends on the textural, structural, surface, and chemical properties of an active catalyst, combined with an appropriate oxidant. Other methods of oxidative desulfurization involve irradiation techniques, ultrasonic methods, direct photo-oxidation, and photocatalytic, electrocatalytic, and plasma techniques [44,61]. Oxidation is accompanied by liquid extraction to separate the oxidized components because of their enhanced polarities. The efficiency of extraction is influenced by solvent polarity, boiling point, and freezing point, as well as surface tension. In addition to common water-soluble polar solvents, ionic liquids are employed to extract S-compounds directly or after they have been oxidized. Recently, a combination of extractive and oxidative desulfurization called extractive catalytic oxidative desulfurization (ECODS) has gained recognition as a technique for producing ultra-low sulfur clean fuels, making using of ionic liquids as extractants and H_2_O_2_ as the oxidant, easily converting organosulfur compounds to sulfoxides and sulfones [62]. Issues associated with ODS, such as lack of oxidant selectivity, unwanted side reactions, undesirable residual S-containing aromatic moieties due to incorrect choice of extraction solvent, and high cost, are carefully weighed against the benefits linked with this process that cannot be achieved in conventional HDS process. For more information, we direct our readers to some new reviews that have appeared in literature focusing on ODS catalysts for clean environment, ODS of heavy oils with high sulfur contents, ODS using heteropolyacid-based catalysts, and ultrasound assisted ODS of hydrocarbon fuels [63,64,65,66,67].

### 3.3. Adsorptive Desulfurization (ADS)

There is a direct link between microporosity and adsorption capacity for efficient desulfurization. There has been a quest for materials with pores larger than 10 Å and adsorbents, such as zeolites, activated carbon, metal-organic frameworks (MOFs), aluminosilicates, and ZnO, have been tested for capturing and eliminating organosulfur compounds present in high value fuels. In addition to having excellent porosity, an ideal adsorbent has high available surface area, high selectivity, good surface chemistry, and recyclability for efficient removal of S-compounds. ADS can proceed through two main approaches: physical adsorption, a less energy intensive pathway where the S-based compounds are not chemically modified on separation; and reactive adsorption that offers benefits of both catalytic HDS and physisorption. A chemical reaction taking place between S-based moieties and solid adsorbent in the latter approach releases sulfur in the form of H2S, Sox, or elemental S. For practical industrial applications, more studies and engineering at a molecular scale is required to adjust the size of host pores to match the guest size and to avoid any steric hindrance between guest molecules.

### 3.4. Biodesulfurization (BDS)

With advancement in biotechnology, green processing of fossil fuels has become possible, and microorganisms with an affinity for sulfur are playing an important role in metabolizing organosulfur compounds in fuels. This technology has potential to become cost effective and efficient. Microorganisms, such as *Pantoea agglomerans*, *Alcaligenes xylos-oxidans*, *Rhodococcus erythropolis*, *Mycobacterium*, and thermophilic *Paenibacillus*, have been identified for aerobic BDS, while *Desulfovibrio desulfuricans*, *Desulfomicrobium scambium*, and *Desulfovibrio longreachii* have been for anaerobic BDS. Kirkwood et al. indicated in their studies that specificity for sulfur and metabolic pathways may not be dependent only on sulfur but, rather, on the type of species used [68,69,70]. Metabolism of organosulfur compounds and C–S bond cleavage by bacteria is suggested to proceed either through reduction of C–S bond and oxidation of C–S bond, or through oxidation of C–C bond (Kodama pathway) [71,72]. Although BDS has several advantages over conventional desulfurization processes, it can be uneconomical due to the additional cost associated with culturing the bacteria [73]. Other important drawbacks include phase change (fuel-aqueous phase) and stability of cells in the presence of fuel species.

### 3.5. Other Forms of Desulfurization

British Petroleum had developed and tested alkylation-based desulfurization on light oils, where acid catalysed aromatic alkylation is performed on thiophenic compounds with an aim to upgrade olefinic gasoline by increasing the molecular weight and boiling point of alkylated sulfur compounds, so that their separation becomes feasible [74]. Another simplified form of alkylation is S-alkylation, where thiophenic molecules are made to reaction with methyl iodide and fluoroborate salts of silver, leading to alkylated sulfonium salts which are precipitated and easily eliminated without distillation [75]. However, S-alkylation is not selective in these reagents, owing to their affinity to aromatic hydrocarbons, making this process challenging in carbon rich heavy oils.

C–S and S–S bonds can also be cleaved by chlorinolysis, where chlorine is able to bind at sulfur sites. This process takes place under moderate operating conditions (25–80 °C, atmospheric pressure) in fuels that can be easily homogenized with chlorine and which are corrosion resistant in the presence of chlorine. In order to remove impurities, additional steps comprising hydrolysis, oxidation of sulfur, and several aqueous and caustic washes are usually performed after chlorination.

Supercritical aqueous desulfurization or supercritical water desulfurization (SCW) has been shown to break C–S bonds in non-aromatic sulfur molecules through free radical pathway. However, some studies have shown that SCW independently cannot desulfurize fuels when not in the presence of H_2_ and conventional HDS catalysts. There are indeed some benefits associated with SCW, such as increase in liquid yield, precipitation of sulfur saturated compounds, and H_2_ generation through water gas shift. To discuss further on industrially applicable methods for desulfurization is beyond the scope of this review; thus, for more information, we direct readers to some excellent reviews on this topic provided by Javadli et al. [61] and Srivastava et al. [44], as well as advances in biodesulfurization by Sadare et al. [73].

## 4. Mesoporous Silica and Mesoporous Silica Nanoparticles in Desulfurization

### 4.1. Photocatalytic MSs

**Figure 1:**
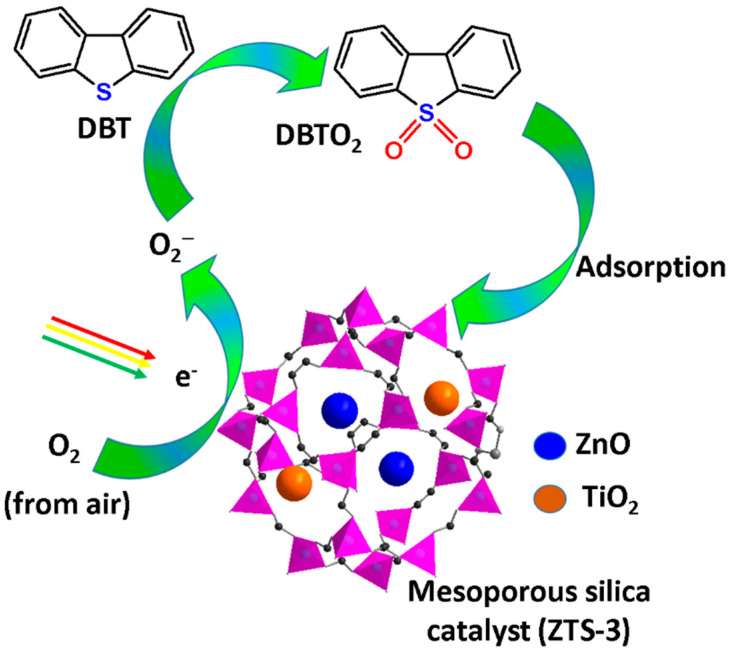
Photocatalytic desulfurization mechanism of ZnO/TiO_2_–SiO_2_ (ZTS-3) catalyst under solar light [78].

Photocatalytic adsorption desulfurization (PADS) has emerged as a form of oxidative desulfurization method due to the low cost, high stability, and recyclability of photocatalytic materials [76,77]. Solar power is capable of exciting the photocatalysts and generating holes that are great oxidants. Certain aromatic sulfurous molecules, including DBT, DBTO (dibenzothiophene sulfoxide), and DBTO_2_, can be oxidized in the presence of free radicals, such as ^•^OH and ^•^O_2_^−^, and PADS is useful in deep desulfurization requirements. Recently, Zhou et al. reported on the use of mesoporous ZnO/TiO_2_–SiO_2_ (ZTS) as a photocatalyst and adsorbing material towards organic sulphides [78]. ZnO is known for its high photocatalytic activity and has a bandwidth of 3.2 eV (380 nm) [79]. Previous studies have shown that performance of ZnO can be enhanced dramatically in the presence of TiO_2_ by inhibiting the recombination of electrons and holes, extending their lifetime and enlarging the photoresponse range [80]. Inspired by these findings, Zhou et al. implemented these studies for synthesising effective desulfurizing agents [78]. ZnO were chosen as active sites in the mesoporous TiO_2_–SiO_2_ catalyst, and the TiO_2_–SiO_2_ precursor materials were synthesized hydrothermally in the presence of triblock copolymer (P123) as a template, so as to tune the pore size. ZnO was incorporated using impregnation techniques, and the ratio of Si/Ti was adjusted in order to achieve optimized adsorption PADS capacity. The authors showed that ZTS-3 with Si/Ti = 3 exhibited the best photocatalytic desulfurization activity. The DBT conversion rate was found to be 97% in 4 h, and maximum adsorption of DBT over ZTS-3 was 47 mgS per gram of catalyst used and the adsorption rate was much higher than mesoporous TiO_2_–SiO_2_-40 (13.7 mg S/g-cat) [81]. The photocatalytic activity of ZTS was attributed to the heterojunction formed through the interactions of ZnO with TiO_2_, leading to the expansion of sunlight absorption, enhancing the efficiency of charge separation, and inhibiting electron–hole recombination. Interestingly, this desulfurization could proceed without involving extra oxidants, such as hydrogen peroxide, O_2_, or organic oxidants, making it a cost-effective process that exhibits high PADS performance and excellent adsorption capacity. As shown in Figure 1, the photocatalytic mechanism is suggested to proceed through the formation of ^•^O_2_^−^, which is capable of oxidizing DBT to DBTO_2_ [78].

TiO_2_ in varying structures and morphologies are among the most promising photocatalytic agents for different applications, such as fuel desulfurization and degradation of contaminants in various systems. In 2017, Meizhen et al. prepared a photocatalytic TiO_2_-modified bimodal mesoporous silica structure (TiO_2_/BMMS) and compared its desulfurization of dibenzothiophene efficiency to that of the mono-modal mesoporous (TiO_2_/SBA-15) catalyst and pure TiO_2_ [82]. They found that the photocatalytic activity and desulfurization efficiency of TiO_2_/BMMS was the highest, achieving a desulfurization rate of 99.2%, followed by TiO_2_/SBA-15 and then pure TiO_2_. In 2014, Zaccariello et al. found that TiO_2_ nanoparticles confined within mesoporous silica nanospheres exhibited enhanced photocatalytic activity due to the added adsorptive effects and thermal stability of the mesoporous silica nanospheres [83].

### 4.2. Redox Active MSNs

**Figure 2:**
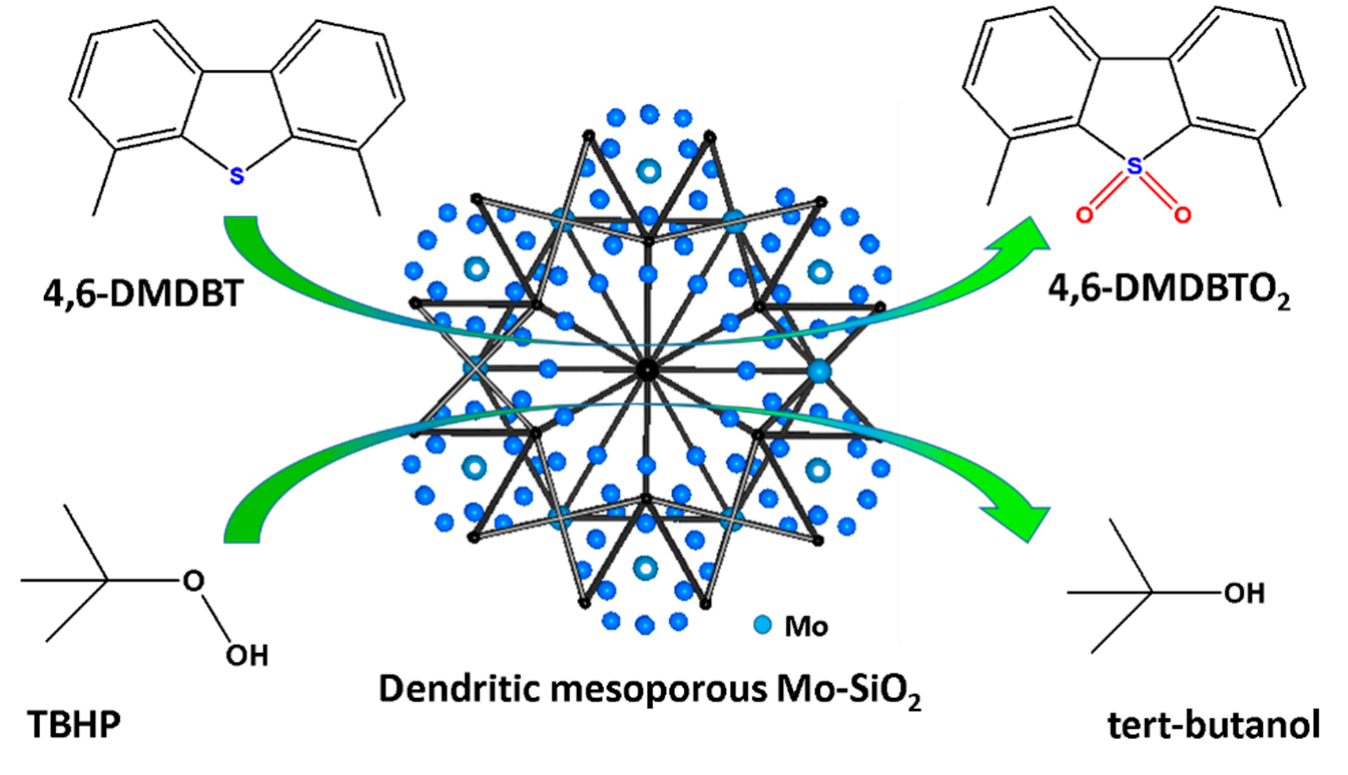
Schematic representation of dendritic mesoporous Mo-SiO_2_ catalyst and its desulfurization mechanism [91].

Conventional desulfurization strategies are associated with issues that hinder their implementation in desulfurization industry, and these problems include high concentration of oxidants, such as hydrogen peroxide, and elongated reaction times [84]. In order to overcome these issues, it becomes essential to formulate suitable catalysts for an eco-friendly and efficient oxidative desulfurization process. Polyoxometalates (POMs) belong to a category of transition metal-based oxygenated anionic clusters, which have emerged in recent years as promising materials for homogeneous oxidation desulfurization (HOD) process, owing to their unique structural and chemical characteristics and their redox potential. Advances in nanotechnology has made possible the immobilization of POMs onto solid supports, such as metal organic frameworks (MOFs), zeolites, porous carbons, and porous silica materials [85,86,87,88]. This addition not only imparts stability and reusability to the POMs but also enhances their catalytic activity towards refractory S-based molecules. Specially designed dendritic mesoporous silica nanospheres with center-radial oriented large mesopores were reported earlier by Zhao et al. as potential carriers or substrates for development of new functional materials [89,90]. Interestingly, Zhang et al. took advantage of the above materials to design a molybdenum-embedded dendritic mesoporous silica sphere using Stöber approach [91]. As shown in Figure 2, they introduced active molybdenum species with a concomitant use of ionic liquid (IL) as a metal source into the mesopores of dendritic silica spheres (DSSs). The DSSs featured large specific surface areas and high pore volumes and possessed a highly dispersed molybdenum species. The authors explored optimum synthetic conditions and characterized ODS products using gas chromatography–mass spectrometry GC-MS analysis. The judiciously designed catalyst showed rapid and high catalytic activity in oxidizing 4,6-DMDBT, and the catalyst could be easily separated from a heterogeneous mixture after fulfilling its role. Under optimal conditions, elimination of 4,6-DMDBT could reach 100% in 40 min, and a low dosage of oxidant was required. The recycling performance of the DSS revealed that they could be reused nine times without an appreciable decrease in catalytic activity [91].

**Figure 3:**
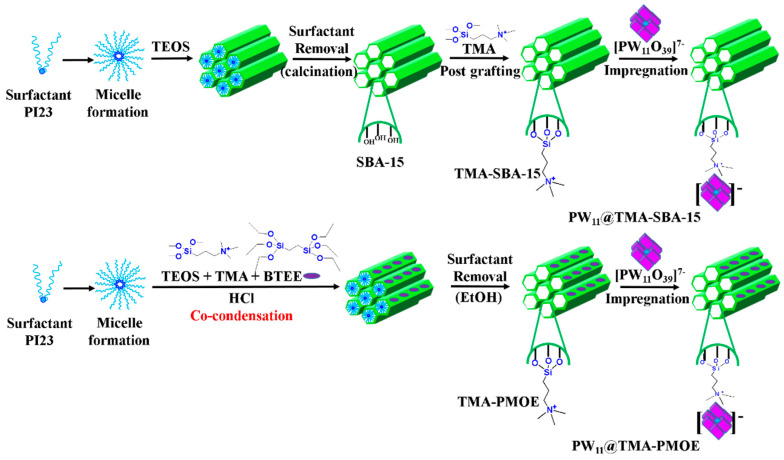
Synthetic strategy to prepare Keggin’s polyoxometalate anion incorporated PW_11_@TMA- (SBA)-15 and PW_11_@TMA-PMOE [101].

Exemplary performance of monolacunary [PW_11_O_39_]^7-^ Keggin polyoxometalates in oxidative reactions [92,93,94,95,96] has inspired a few researchers to explore their performance in ODS process, too [97,98,99]. For example, Ribeiro et al. have worked extensively on Keggin-type materials, including PW_11_ embedded on amine decorated SBA-15 (PW_11_@aptesSBA-15 and PW_11_@tbaSBA-15), and on cationic tma functionalized mesoporous silica supports for the desulfurization of model and real diesels (Figure 3) [100]. Oxidative desulfurization studies on amine functionalized SBA-15 supports revealed high performance in case of PW_11_@aptesSBA-15, which could completely desulfurize simulated diesel in solvent-free conditions with half the oxidant amount (H_2_O_2_/S = 4), whereas it showed 83.4% efficiency in case of real unthread diesel (in a biphasic system, 1:1 diesel/acetonitrile) with a recycle capacity for eight consecutive cycles. Their recent work involved impregnation of PW_11_ on cationic group (*N*-trimethoxysilypropyl-*N, N, N*-trimethylammonium, TMA) functionalized MS supports [101]. Here, two kinds of MS supports were selected (ordered MS, SBA-15, and an ethylene-bridged periodic mesoporous organosilica, PMOE) and two catalysts were prepared, PW_11_@TMA-SBA-15 and PW_11_@TMA-PMOE. Oxidative desulfurization was carried out on simulant diesel under biphasic (1:1 diesel/acetonitrile) and solvent-free conditions. A remarkable desulfurization performance was exhibited by the PW_11_@TMA-SBA-15 catalyst, which could achieve ultra-low sulfur levels (<10 ppm) in both biphasic, as well as solvent-free conditions, and could be recycled six consecutive times without appreciable loss of catalytic activity. This promising catalyst was also tested on untreated real diesel provided by Spanish petroleum company, CEPSA (1335 ppm S) under biphasic system and demonstrated 90% desulfurization efficiency for 3 consecutive cycles [101].

Recently, Ribeiro et al. reported on zinc-incorporated polyoxotungstates [PW_11_Zn(H_2_O)_39_]^5−^, PW_11_Zn for ODS of real, as well as model, diesels, where active species were embedded in periodic mesoporous organosilicas (PMOs) composed of walls with ethane-bridges and benzene bridges [102]. These compounds showed high desulfurization efficiency under solvent-free conditions and in the presence of H_2_O_2_ with a H_2_O_2_/S ratio of 4, where ultra-low levels of sulfur could be obtained in just 1 h. PW_11_Zn@aptesPMOE catalyst was further investigated due to its robust nature for sulfur removal of real diesel, and an efficiency of 75.9% could be achieved after 2 h, and the catalyst could be reused.

A heteropolyacid-based adsorbent catalyst was reported by Yuzbashi et al. with an aim to decrease the leaching of active components and to increase the ODS kinetics of DBT present in model diesel fuel [59]. Phosphotungstic acid (HPW) was incorporated in a zirconium-modified hexagonal mesoporous silica (Zr-HMS) to give HPW/Zr-HMS catalyst, and its DBT removal ability was compared with Zr-HMS, HPW-HMS. Zr-HMS showed only 15% S-removal efficiency, whereas the efficiency of the two others was found to be similar; however, an efficiency of 99.4% was observed within 120 min when the composition of prepared catalyst was 20% HPW/Zr-HMS. An increase in the catalyst dosage from 0.03 to 0.05 g drastically increased S-removal efficiency from 10% to 99.4%. In the presence of H_2_O_2_, the model fuel was oxidized in less than 30 min and achieved an efficiency greater than 95% of the 350 ppm DBT. The formulated catalyst was recovered and was reused at least five times, and the leaching of HPW species was inhibited, indicating the promising nature of catalyst for ultra-deep desulfurization process [59].

There have been cases where, instead of using heteropolyacids, silicotungstic acids have been used directly for the preparation of mesoporous tungsten-silica catalysts. For example, Shen et al. reported on the formation of disordered worm-like mesoporous silica catalysts with well dispersed tungsten species using nanocellulose as templates [24]. High surface area of the formed catalysts in the range of 344–535 m^2^/g, their narrow pore size distributions (2 nm to 10 nm), and an optimum Si/W ratio supported high catalytic activity towards DBT and its elimination at 60 °C in just ten min. The catalytic activity towards S-compounds was found to increase in the following order: BT < 4,6-DMDBT < BT. GC–MS analysis of oxidized species showed that the catalyst formulations not only played their role as catalysts but also acted as efficient adsorbents. This catalyst could be recovered and be reused at least five times and still retain its desulfurization efficiency up to 94% [24]. The authors of this report had proposed a mechanism for the oxidation of DBT where the first step involves the adsorption of DBT in the pores of the catalysts, followed by its reaction with active peroxo-tungsten complex, which in turn is a direct consequence of reaction between tungsten species and H_2_O_2_ as oxidant. The interactions between DBT and peroxo-tungsten complex converts DBT to DBTO_2_, which gets adsorbed in the mesopores of the catalyst, with the simultaneous reduction of peroxo-tungsten complex to tungsten oxide.

**Figure 4:**
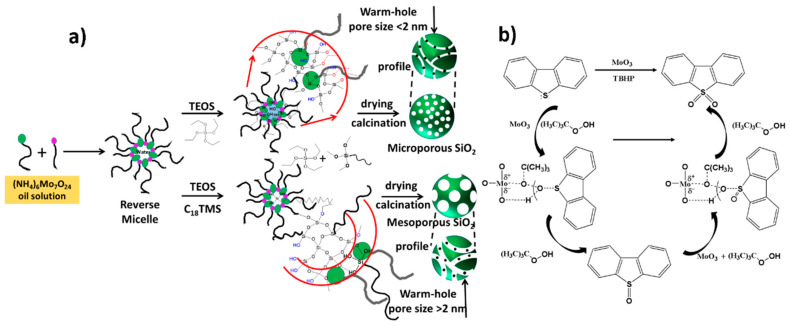
(**a**) Schematic representation for the formation of subnano-MoO_3_/ultrasmall mesoporous silica nanoparticles (UMSN); (**b**) catalytic mechanism of MoO_3_ catalyzing dibenzothiophene (DBT) oxidation in the presence of TBHP (tert-butyl hydro peroxide) [106].

With advances in nanoscience, unique properties have been observed for particles with an average diameter lying in the range of 0.1 nm to 1.0 nm. This size range sets them apart from their nano counterparts, and some refer them as subnanoclusters [103,104]. A large number of catalysts are being developed using metal oxide subnanoclusters, and many materials have shown promising catalytic behavior for a large number of organic reactions. Along the same lines, the size of mesoporous silica materials is also decreasing, and new ultrasmall MSNs (UMSNs) with particle size less than 25 nm are being developed in order to reduce mass transfer resistance and enhance the catalytic activity. Some researchers, like Wang et al., reported on the synthesis of subnano-MoO_3_ supported on ultrasmall MSN particles (~14 nm) with a peculiar “raisin-bun” structure, using reverse microemulsion technique (Figure 4a) [105]. This hybrid catalyst, subnano-MoO_3_/ultrasmall MSN (UMSN), was then used for ODS of a model diesel comprising of benzothiophenes, where they were oxidized to DBTO_2_ with a 100% efficiency within 15 min (Time of flight, TOF of ODS: 53.3/h) (Figure 4b) [106]. Percentage of catalyst, reaction time, and temperature influenced the DBT conversion; however, the percentage of oxidant did not play a significant role. Surprisingly, performance of nano-MoO_3_/UMSN tested under similar operating conditions showed a DBT to DBTO_2_ conversion of only 25.7% (TOF was 10.2/h), proving that subnano-MoO_3_ was more active than nano-MoO_3_. It is well known that smaller size generates high catalytic active sites with higher ratio of surface to bulk atoms; however, an 80% efficiency drop was attributed to the higher binding energy associated with subnano particles (1.1 eV higher than bulk), appearing as a result of lowered core-hole screening existing in small clusters. This indicates that electronic properties show significant variation with size and can show unusual response at subnano level.

**Figure 5:**
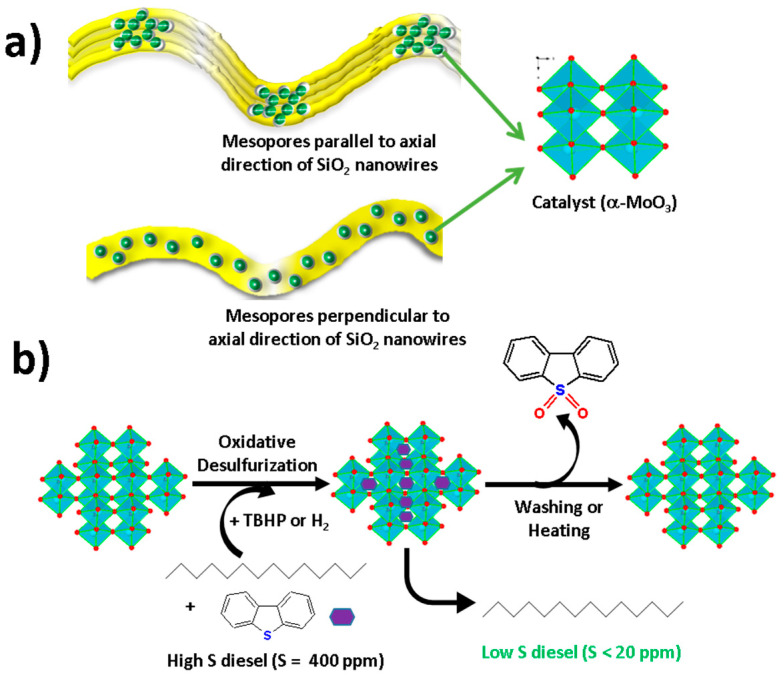
(**a**) Structure of mesoporous silica nanowires with mesopores aligned parallel or aligned perpendicular to the axial direction of nanowires; (**b**) desulfurization mechanism showing reaction of DBT unit with catalytic species and removal of DBTO_2_ molecules after oxidation [107].

Development of 1D silica fibers with diameters of about 50 nm or less and their transformation into materials with unique pore architectures, especially with pores aligned perpendicular to the direction of fibers, has become of paramount importance for materials scientists. Aiming to improvise catalytic and sorption properties of silica materials, Dou and Zeng developed interconnected mesoporous silica nanowires with a 3D network having shallow wormhole channels aligned perpendicular to the axis of nanowires using a simple synthetic strategy, where TEOS hydrolysis took place under basic conditions (Figure 5) [107]. They explored synthetic parameters, including the effect of cosolvents, surfactants, alkalinity, duration of reaction, and optimum aging temperatures, to get an insight on the formation mechanism. α-MoO_3_ species were immobilized on the interconnected mesoporous SiO_2_ nanowires to form a Mo/mSiO_2_ catalyst−adsorbent system and was examined in ODS of model diesels.

Although, in these studies, the diameter of Mo/mSiO_2_ nanowires was significantly reduced and reached in the range of 10–20 nm, the authors were able to increase the final dimensions of the catalyst to a micron level, allowing easy recovery and separation after being used. The BET surface area of Mo/mSiO_2_ reduced from 852 to 503 m^2^/g on increasing the Mo loading from 2 to 15%. The recipes containing 5 to 10% Mo showed highest conversions of DBT, owing to an optimum balance between total working catalyst and effective surface area. For a 10% Mo/mSiO_2_ nanowire recipe, a DBT conversion efficiency remained as high as 95.4% in the first 30 min, even after seven cycles, and the sulfur concentration was 33 ppm after the seventh run.

### 4.3. Ionic Liquid MSN Catalysts

Ionic liquids (ILs) have emerged in the past few years as green solvents and catalysts, thanks to their unique properties that include high thermal stability, feasible design, and synthesis, as well as a wide liquid range [108]. These features allow dissolution of several catalysts in the ionic liquids providing a biphasic liquid system beneficial not only in esterification [109] and fructose dehydration [110] but also in oxidation of sulphides [111,112]. However, necessity for large amounts of ILs and difficult catalyst recovery makes it challenging to apply them in wide range of organic reactions, except when these ILs can be heterogenized. Therefore, many attempts have been exerted towards fabrication of “supported ionic liquid catalysts” (SILCs) that can immobilize homogeneous ILs on appropriate host materials, which can range from silica-based materials and MOFs to carbon nanotubes [113,114,115,116,117,118]. Advantages associated with use of mesoporous silica have compelled researchers to use impregnation and grafting methods to prepare SILCs, but these efforts are accompanied by other issues, like reduction in surface area of supports, leaching of active components, or confinement in the degree of freedom of ILs [119,120]. These limitations can be overcome by incorporation of functionalized ILs in the grafting matrix, by performing one pot hydrothermal synthesis in architectural supports, and by removing templates in subsequent reactions [121,122,123]. In our opinion, it is worth discussing examples where ionic liquids have been immobilized on mesoporous silica materials to be used for desulfurization process.

A few years ago, Gu et al. reported on the synthesis of hybrid mesoporous silica material using task-specific ionic liquid (TSIL) templating strategy, where POM-based IL [(n-C_8_H_17_)_3_-NCH_3_]_2_ [W_2_O_3_(O_2_)_4_] (termed as T_8_W_2_O_11_) was used as template [124]. The authors blended the amphiphilic T_8_W_2_O_11_ with silicate source tetraethyl orthosilicate (TEOS). Silicate condensation lead to the entrapment of T_8_W_2_O_11_ species, and subsequent calcination eliminated the organic cations, leaving behind a functionalized mesoporous silica framework. Catalytic activity of the resulting product was tested in oxidative desulfurization process, where 99.6% efficiency was observed in the case of DBT sulfur removal using low catalyst dose and without using organic solvents. The efficiency of catalytic oxidation of organosulfur compounds decreased in the following order: DT > 4,6-DMDBT > DBT > BT. The efficiency of the catalyst could be retained, even after recycling eight times [124].

**Figure 6:**
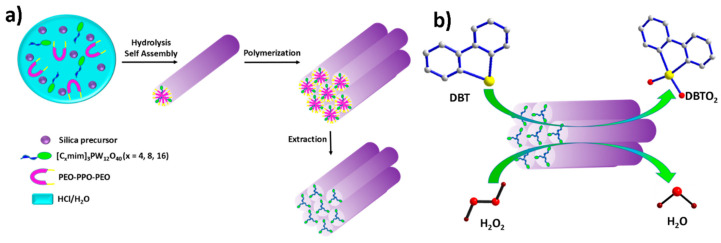
(**a**) Fabrication of ordered mesoporous silica encapsulating polyoxometalates (POMs)-based ionic liquids (IL); (**b**) possible desulfurization mechanism for the oxidation of DBT to DBTO_2_ with concomitant transformation of hydrogen peroxide to water [125].

This work was followed by Zhang et al., who reported encapsulation of polyoxometalate-based ILs with the formula [C_x_mim]_3_PW_12_O_40_ (C_x_-IL, x= 4,8, and 16) into the pores of ordered mesoporous silica through a one-pot hydrothermal reaction (Figure 6a) [125]. The as-prepared catalytic material (C_x_-IL@OMS) was systematically characterized using multiple chemical and physical techniques, proving its high specific surface area and uniform dispersion in silica supports. These catalysts were employed in the desulfurization of organosulfur compounds, and [C_4_mim]_3_PW_12_O_40_@OMS was a winner among the series (Figure 6b). It showed high catalytic activity, high stability, and could be recycled multiple times. When compared with other IL@OMS, catalytic oxidation of DBT decreased in the following order: C4-IL@OMS (99.5%) > C8-IL@OMS (76.7%) > C16-IL@OMS (30.5%). High performance of C4-IL@OMS could be ascribed to its large surface area providing highly exposed active sites for oxidation of DBT and short IL chain imparting low steric hindrance and enough platform for peroxide species to interact with DBT molecule. Interestingly, catalytic oxidative desulfurization efficiency of pure C4-IL (S removal:12.7%) was much lower in comparison with C4-IL@OMS (S removal:99.5%). Additionally, even after recycling seven times, the removed sulfur in case of C4-IL@OMS exhibited an impressive 93% efficiency [125].

**Figure 7:**
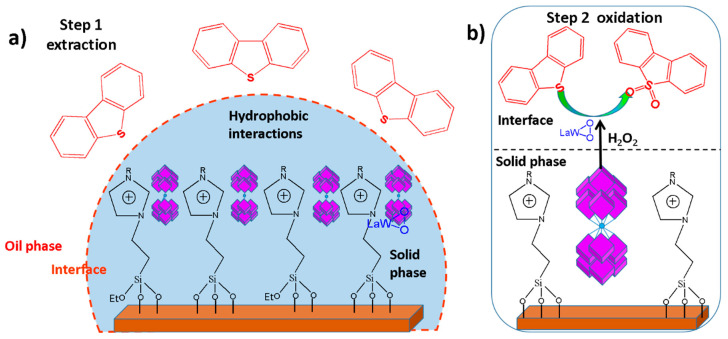
Schematic illustration for the highly selective and efficient removal of sulfur species using LaW_10_/IL-SiO_2_ nanocomposites. (**a**) Extraction of the substrate by ionic liquid, (**b**) catalytic oxidation of DBT to DBTO_2_ by LaW_10_ in the presence of H_2_O_2_ (R = −C_8_H_17_) [128].

Prior studies have also shown lanthanide-based POM clusters Na_7_H_2_LaW_10_O_36_·32H_2_O (LaW_10_) as promising catalysts for ECODS process [126,127]. However, new materials are required to make ECODS more efficient and highly selective in terms catalyst separation, recovery, and reusability. As shown in Figure 7, Chen and Song impregnated LaW_10_ on dihydroimidazolium (ionic liquid) modified mesoporous silica supports, forming LaW_10_/IL-SiO_2_ [128]. The new catalyst exhibited deep desulfurization of several organosulfur compounds, including DBT, BT, and 4,6-DMBT, under mild operating conditions and in less than 30 min.

Ultra-deep desulfurization (<100 ppm) could be achieved for DBT in just 1 min for small batches, whereas scaled-up studies (1 litre model oil with S content of 1000 ppm) showed 100% sulfur removal in 25 min and at 70 °C. Additionally, the process was cost-effective as reusability of catalyst did not require simultaneous use of ionic liquid each time, recovery of LaW_10_/IL-SiO_2_ catalyst was easy, and it could be reused at least ten times without degrading its efficiency, making it a promising material for ultra-low sulfur fuels [128].

Although some mesoporous silicas, such as MCM-41 and SBA-15, possess uniform pore size and a large internal surface area, their small pore size at times can lead to blocking and collapsing of pores during the desulfurization process, eventually giving poor recycling performance. In order to overcome these challenges, three-dimensional ordered macroporous (3DOM) materials with well-connected pores, large pore size sufficient to catalyze most fuel related organosulfur compounds, better mass transfer properties, and high surface areas are being developed [129,130]. Silica-based meso-macroporous materials (H_3_PW_12_O_40_/SiO_2_) synthesized via polystyrene colloidal crystal template and using evaporation-induced self-assembly (EISA) have been reported by Lei et al. and applied in the ODS process [131,132]. In both cases, hierarchically meso/macroporous catalysts exhibit superior catalytic activity than their purely mesoporous and macroporous counterparts. This enhanced performance can be attributed to the larger mesoporous specific surface area, peculiar hierarchically meso/macrochannels structural characteristics and shorter length of mesoporous channels that facilitate mass transfer of precursors and products. Recently, Chen et al. reported on a POM-based ionic liquid supported 3DOM silica (IL-3DOM SiO_2_) as a catalyst for heterogeneous oxidative desulfurization [58]. The catalyst exhibited high porosity, as well as, a high specific surface area. This catalyst was prepared using the colloidal crystal template assembled from polymethylmethacrylate (PMMA), having a mean diameter of approximately 240 nm. The 3D crosslinked structure of catalyst allows guest diffusion, capture, and exposure of the active species. Sulfur removal efficiency of an IL-3DOM SiO_2_ catalyst in the absence of H_2_O_2_ was only 5.7%, while it was 100% in its presence, indicating the ability of 3D crosslinked structure to effectively adsorb organo sulfur molecules and essential presence of tungsten-based IL to activate H_2_O_2_ to oxidize S-compounds. The desulfurization performance of IL-3DOM SiO_2_ catalyst decreased in the following order: 4-MDBT > DBT > 4,6-DMDBT > 3-MBT > BT. Additionally, even after recycling 17 times, the sulfur removal efficiency could still reach 94% in the absence of a regeneration process [58]. The mechanism of DBT desulfurization by IL-3DOM SiO_2_ is shown to proceed, firstly via adsorption of DBT in the macropores and then its reaction with peroxo species formed in turn from the combination of POM-IL with H_2_O_2_. Subsequent steps involve the oxidation of DBT to the corresponding sulfones (DBTO_2_), their precipitation, and presence in the catalyst phase. Towards the end of cycle, the peroxo species convert into active species, which further combine with H_2_O_2_, forming species for the next cycle [58].

Compared to conventional mesoporous silicas, such as MCM-41 or SBA-15, the reports on the corresponding nanoparticles as adsorbents for desulfurization are very limited. Last year, Mirante et al. reported on composite materials prepared through the immobilization of Keggin polyoxomolybdate [PMo_12_O_40_]^3-^ anion on MCM-48 type mesoporous silica nanoparticles functionalized with surface-tethered tributylammonium (TBA) groups [133]. The composite catalyst PMo_12_@TBA-MSN had high stability and was highly efficient in the oxidative desulfurization (ECODS) of a diesel simulant in the presence of H_2_O_2_ (oxidant) and [BMIM]PF_6_ (IL and solvent). The shorter channels of MSNs and cubic mesoporous framework of MCM-48 nanoparticles with branched networks seem to allow mass transfer in comparison to conventional porous silica materials with unidirectional structures. The ECODS studies were carried out on a model diesel composed of BT, DBT, 4-MDBT, and 4,6-DMBT in n-octane with a total sulfur content of 2016 ppm. For the first ECODS cycle, the efficiency of PMo_12_@TBA-MSN was high and comparable to the homogeneous catalyst PMo_12_, where desulfurization could be achieved in 2 h. However, PMo_12_ could not be reused in the subsequent cycles, while the hybrid catalyst PMo_12_@TBA-MSN exhibited higher stability and could be reused, along with ionic liquid, several times. The impregnation of PMo_12_ on MSNs proved to be beneficial in enhancing the stability of polyoxomolybdate and in obtaining not only a robust and effective catalyst but also a recyclable material [133].

In comparison to polyoxometalates (POMs), Keggin-type heteropolyacids (HPAs) have shown high catalytic activity for oxidation studies, owing to their high chemical resistance and mechanical robustness, making them suitable for desulfurization reactions as mentioned earlier, when they were combined with meso/macroporous silica [58]. Heteropolyacids have octahedral MO_6_ units, and their lowest unoccupied molecular orbitals (LUMO) are mainly the non-bonding metal orbitals, allowing easy participation in redox reactions [134,135]. A heterogeneous catalyst (HPMo-IL/SBA-15) was reported by Xiong et al. via impregnation of phosphomolybdic acid on the IL functionalized SBA-15 and used for the ODS process [136]. The hydrophilicity of SBA-15 was conquered by functionalization with imidazole-based IL, and the heterogeneous HPMo-IL/SBA-15 catalyst exhibited good wettability for the model oil while providing sufficient active sites for the access of organosulfur compounds. The catalytic system involved the use of H_2_O_2_ with a H_2_O_2_/sulfur mole ratio of 2, which is much less in comparison to other desulfurization systems, and, in the presence of 0.05HPMo-IL/SBA-15, a S-removal efficiency of 90% could be achieved. High catalytic performance of HPMo-IL/SBA-15 is attributed to its (a) high surface area and good IL dispersion; (b) uniform pore channels allowing efficient mass transfer; and (c) high wettability towards model oil [136].

### 4.4. Magnetic MS Catalysts

**Figure 8:**
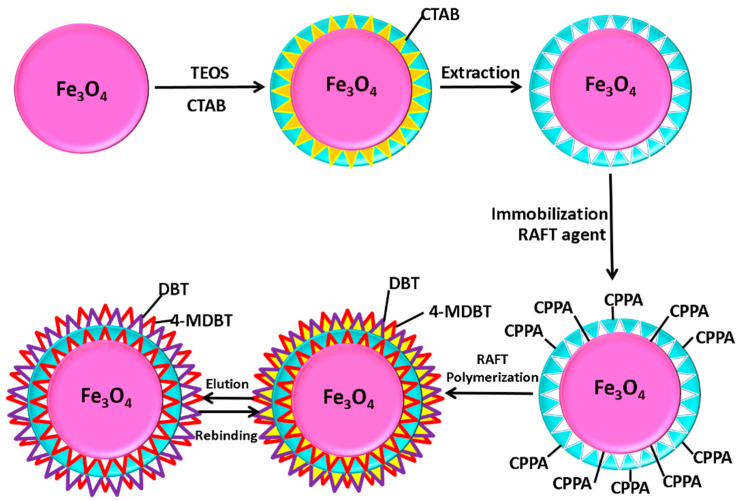
Schematic illustration for the preparation of functionalized magnetic mesoporous silica (Fe_3_O_4_@mSiO_2_@DT-MIP) covered with double template molecular imprinted polymer (MIP) [141].

Catalytic materials synthesized using surface molecular imprinting techniques have shown promise for deep desulfurization reactions. Surface imprinted polymers are beneficial in terms of high guest inclusion rates, reduced mass transport resistance, rapid adsorption kinetics, and feasible template extraction. Additionally, as far as complex fuel samples are concerned, magnetic separation can help to separate impurities and purify the process by adsorption of organosulfur compounds on magnetic catalysts. If molecular imprinted polymers (MIPs) are embedded with magnetic nanoparticles or porous materials doped with magnetic nanoparticles, these adsorbents can be separated in the presence of applied magnetic field [137,138]. Few researchers, including Li et al. and Men et al., have applied this strategy to prepare surface imprinted core-shell magnetic beads and magnetic MIPs for the adsorption of template molecules and their separation from the matrix [139,140]. However, there are only few reports of this strategy used for desulfurization of fuels. With an aim to develop efficient materials for deep-desulfurization of fuels, Wang et al. reported on the synthesis of molecularly imprinted polymer (MIP) coated magnetic mesoporous silica, Fe_3_O_4_@mSiO_2_@DT-MIP, using double-template strategy for desulfurization of DBT and 4-MDBT in model and real gasoline fuels (Figure 8) [141]. The adsorption studies revealed that Fe_3_O_4_@mSiO_2_@DT-MIP followed pseudo-second-order kinetics in the case of DBT and 4-MDBT, with an adsorption amount of 104.2 mg g^−1^ for DBT, whereas it showed an amount of 113.6 mg g^−1^ towards 4-MDBT, respectively. When the performance of molecular imprinted polymer was compared with non-imprinted polymer (NIP), it revealed that Fe_3_O_4_@mSiO_2_@DT-MIP had higher binding ability and significant recognition capacity for DBT and 4-MDBT, and it could be regenerated and reused at least eight times more than Fe_3_O_4_@mSiO_2_@NIP. When adsorption experiments were carried out on real fuel (92#gasoline), Fe_3_O_4_@-mSiO_2_@DT-MIP catalyst was able to reduce the amounts of DBT and 4-MDBT by at least by 69% [141].

The catalytic activity of materials is of prime importance in biphasic reaction of oil and water and most hydrophilic catalysts can be hindered on interactions with hydrophobic aromatic sulfur compounds. Ionic liquids can come to rescue in these situations by providing active sites and also making the carriers hydrophobic. An ideal ionic liquid has optimum length of carbon chain which does not cause steric hindrance and easy leaching of active species. Inspired by the superhydrophobic lotus leaves, it can be concluded that rough surfaces on carriers can change their wettability, and Zhao et al. demonstrated this by synthesizing core-shell mesoporous silica structures which had cauliflower mimicking morphology [142]. These materials were modified using kinetic-controlled interface co-assembly to produce magnetic mesoporous microspheres (MMSs) which acted as supports for POM-based short chain ionic liquids (([(C_4_H_9_)_3_NCH_3_]_3_PMo_12_O_40_). The contact angle measurements substantiated the wettability of these materials and their desulfurization capacity, which in turn could be regulated by their surface morphology. The catalyst, ([(C_4_H_9_)_3_NCH_3_]_3_PMo_12_O_40_/RS-MMS) was used for the ODS of diesel in the presence of H_2_O_2_ as an oxidant and showed highest catalytic activity towards most refractory sulfur compounds.

A model oil consisting of 4,6-DMDBT as a representative was chosen to investigate the desulfurization efficiency of POM incorporating IL and IL/RS-MMS, and the results revealed that IL showed only 16.3% sulfur removal efficiency, whereas IL/RS-MMS showed 98.3% sulfur removal efficiency at equal time intervals and the same temperatures, which may be attributed to high surface area and better dispersion of active components in IL/RS-MMS catalyst. Sulfur removal followed the order: DBT > 4-MDBT > 4,6-DMDBT, and the effect of ODS reaction temperature for 4,6-DMDBT by IL/RS-MMS revealed an increase in desulfurization efficiency from 41.1% at 40 °C to 98.3% at 60 °C and a reduction in sulfur concentration in model oil to as low as 3.4 mg kg^−1^. When ODS activity of smooth surface (SS) catalysts were compared to those with superhydrophobic rough surfaces, only the catalysts with suitable amphiphilicity displayed good catalytic activity. The recyclability of ODS of 4,6-DMDBT using a IL/RS-MMS catalyst showed that the catalyst could be recovered and be reused at least five times [143].

There have been reports on using triblock copolymer templating strategy to synthesize 2D-hexagonal mesoporous silica structures under acidic conditions [26]. Recently, this method has also been used to create POM-IL-incorporated mesoporous silica nanocomposites (PMS) for desulfurization of fuels. For example, Zhang et al. used triblock copolymer P123, PMO-based IL ([C_16_mim]_3_PMo_12_O_40_), and TEOS to synthesize PMS, which was further impregnated with iron oxide nanoparticles using an equivalent-volumetric ultrasonic impregnation method to form magnetic PMS (MPMS) [26]. The catalysts were used desulfurize and remove multiple refractory sulfur compounds (BT, 3-MBT, DBT, 4-MDBT, 4,6-DMDBT) and showed double-edged influence due to iron oxide, where the catalysts were superparamagnetic and were easily attracted by external magnets, laying the platform for magnetic fixation in the recovery steps. In particular, the 0.5-MPMS recipe exhibited excellent catalytic activity, and the desulfurization performance displayed the following order: DBT > 4-MDBT > 3-MBT > 4,6-DMDBT > BT. Furthermore, using this recipe, a desulfurization efficiency of 94% could be achieved, even after ten cycles, making it, so far, the highest recycled compound and a potential candidate for industrial applications [26].

### 4.5. Spinel Embedded Silicoaluminophosphate Catalysts

**Figure 9:**
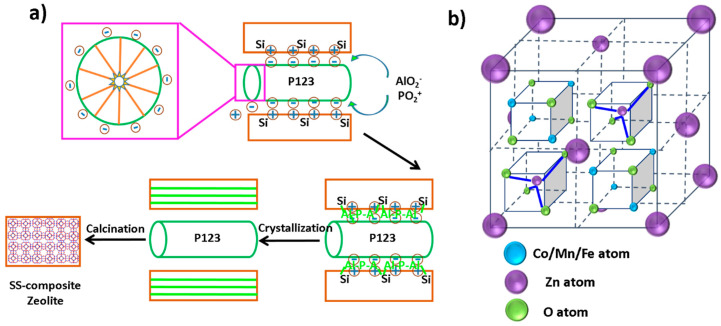
Schematic illustration of (**a**) synthesis of silicoaluminophosphates (SAPO)-34@as-synthesized SBA-15 (silica sphere (SS) composite zeolite) using triblock copolymer (P123) templates; (**b**) ZnCo_2_O_4_ structure [152].

Spinel phase oxides with the formula AB_2_O_4_ (A and B are metals) are known for their high stability and diverse framework architectures. In particular, Zn-based spinel adsorbents (ZnB_2_O_4_) with periodical [B_4_O_4_]^4−^ cubical secondary building units have been reported to considerably subdue vaporization of zinc atoms during hot gas desulfurization when temperatures of the reactors were <600 °C and demonstrates the synergistic effects of ZnO as B_2_O_3_ units on reaction with H_2_S in desulfurization steps [144,145,146]. Additionally, zeolites are high porosity green materials that can prevent sintering of active guest species and have been used in multiple applications, including ion exchange, catalysis, and gas adsorption [147,148,149,150,151]. Silicoaluminophosphates (SAPO) belong to a series of crystalline zeolites, which contain repeating tetrahedral AlO_4_ and SiO_4_ units creating well-defined channels and microporous cavities and have received recent attention in desulfurization process. For example, this year, Liu et al. reported on the fabrication of SAPO-34 zeolites embedded with Zn-based spinels with the formula ZnB_x_B’_2−x_O_4_ (B = Co, Mn, Fe; x = 0–2) with an aim for H_2_S removal from simulated coal gas (Figure 9) [152]. The authors prepared SAPO-34@SBA-15 (SS), SAPO-34@ZSM-5 (SZ), SAPO-34-P123 (SP123), and SAPO-34-PAA (S-PAA) as solid supports for spinel inclusion. SS had a BET surface area of 323 m^2^/g and was used as a representative for multiple examinations. Lattice substitution in B-sites of Zn spinels indicated the stability of ZnCo_2_/SS structure and efficiency in desulfurization process with a high breakthrough S capacity of 138.08 mg/g when compared to other supports impregnated with Fe or Mn. The best recipe for effective desulfurization was found to be 50 wt% ZnCo_2_/SS with a reaction proceeded at 550 °C. With successive regeneration of catalyst, the breakthrough S capacity decreased from 138.08 to 118.69 mg/g, owing to the evaporation of Zn species, generation of highly stable sulfides (ZnS and Co_9_S_8_), and partial sintering of the catalyst [152].

### 4.6. MSN-Carbon Composites

**Figure 10:**
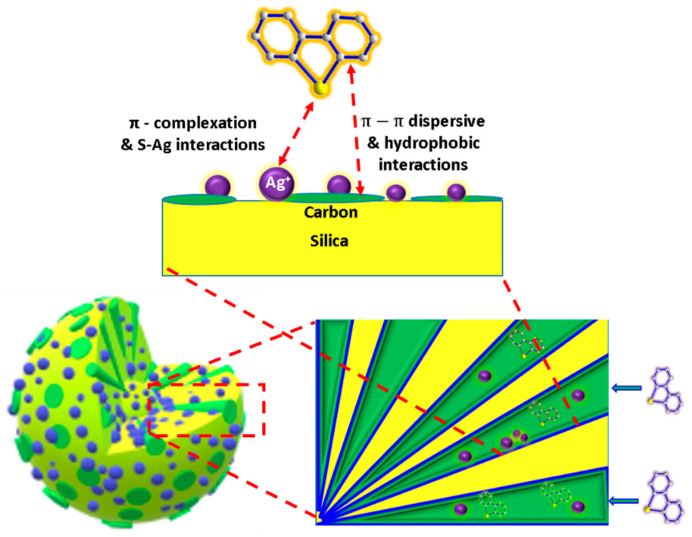
Schematic representation of the synergistic effect of DBT adsorption on dope carbonaceous mesoporous silica supports, Ag/ dendritic mesoporous silica/carbon nanosphere (DMS/CN) structure [155].

In addition to mesoporous silica usage, hydrophobic carbonaceous materials can play an important role in desulfurization due to their high adsorption capacity, high specific surface areas and feasible recovery. Moreover, the wide abundance of carbon materials from biomass to rubber tires makes them lost cost adsorbents [153,154]. Combination of catalytic metal sites on carbonaceous materials embedded on mesoporous silica supports can provide a synergistic effect to the materials for adsorption desulfurization (ADS); however, only a few reports exist in literature on this topic. Recently, Liu et al. reported on the synthesis of monodispersed dendritic mesoporous silica/carbon nanospheres (DMS/CNs) impregnated with Ag ions and used them as sorbents for selective desulfurization of DBT (Figure 10). AgNO_3_ was included in the catalyst through wet impregnation and thermal dispersion techniques. Adsorption dynamics simulation studies indicated that the adsorption rate increased with an increase in the carbon content of DMS/CNs and with a decrease in the particle size of the catalyst, whereas Ag impregnation improved the efficiency of desulfurization, owing to the sulfur and Ag interactions and formation of S–Ag bonds and π-complexation with thiophene rings present in DBT. When compared to non-impregnated S-100-HC (adsorption capacity, 4.02 mg S/g (no toluene), 2.71 mg S/g (toluene)), Ag/S-100-HC was a promising candidate giving an equilibrium adsorption capacity of 6.88 mg S/g without using toluene and 4.22 mg S/g in the presence of toluene. Its enhanced sulfur selectivity could be attributed to its ordered dendritic mesoporous silica/carbon nature with large center-radial mesoporous channels, providing easy access to active metal sites and a synergistic effect between carbonaceous species and embedded silver ions [155].

### 4.7. Silica Gels

High porosity, low cost, and wide availability of silica gels compared to well-ordered MS frameworks have also motivated researchers to explore these materials for desulfurization of fuels. Although few in number, some interesting examples exist in literature where efficiency of silica gel has been explored. For example, Song and co-workers reported on desulfurization of diesel fuel using silica gel impregnated with 5.0 wt% of undisclosed metal species, and a reduction in S content was demonstrated using GC chromatographic analysis by studying the composition of fuel before and after treatment [156]. The authors claimed that JP-8 fuel was used in further studies, and similar results were obtained. Molecular orbital calculations were performed on sulfur rich species, like thiophene, BT, and DBT, and they revealed the presence of HOMO (highest occupied molecular orbital) on sulfur atoms. These studies indicate direct interactions of HOMO orbitals with the LUMO (lowest unoccupied molecular orbital), belonging to the active species present in silica gel, are responsible for selective elimination of sulfur rich molecules in fuel. Wang et al. reported on Ni-heteropolyacids supported on silica gel and used them for removal of thiophene, alkyl thiophene, BT, and DBT under Ultrasound and Ultraviolet irradiation conditions [157]. Soon after this report, Zheng et al. reported on the entrapment of polyoxometalate precursor ([C_16_H_33_(CH_3_)_2_NOH]_3_{PO_4_[WO(O_2_)_2_]_4_}) in the channels of silica gel and used this heterogenous catalyst to oxidize DBT in the presence of hydrogen peroxide [158]. A few years ago, Xun et al. reported on ionic liquid catalysts ([Bmim]FeCl_4_) embedded in silica gel, and various conditions were investigated for the removal of DBT using the catalytic oxidative desulfurization process, where the desulfurization efficiency was over 90%, even after recycling the catalyst six times [159]. This study marked a new era for the emergence of ionic liquid supported silica gels and interesting examples were reported by Zhao et al. and Safa et al. The former study reported on using ether functionalized ionic liquid/silica gel catalysts to adsorb SO_2_ and the adsorption capacities ranged from 2.621 mol of SO_2_/mol for [C_3_O_1_Mim][H_3_CSO_3_] to 3.453 mol of SO_2_/mol for [C_7_O_3_Mim][H_3_CSO_3_], indicating an increase in adsorption capacity with elongation in chain length of the cation [160]. The latter study reported on using 1-octyl-3-methylimidazolium hydrogen sulfate ([Omim][HSO_4]_) in the silica-gel matrix for ODS of a model oil composed of DBT and real diesel fuel. The highest DBT removal efficiency of 99.1% was achieved when 17 wt% of ionic liquid embedded catalyst was used, and 75.7% S-removal efficiency was observed in case of desulfurization of the hydrotreated real diesel fuel [161].

## 5. MS Frameworks and Nanomaterials in Desulfurization of JP-8 and JP-5 Aviation Fuel

Fossil fuels are not only used in combustion engines but also as a source of hydrogen for solid-oxide fuel cells (SOFCs) to be used in vehicles, stationary power generators, or military purpose silent watch units [162]. JP-8 jet fuel is a popular fuel for military applications, where it not only functions as a hydrogen source for SOFCs but also to power the aircrafts, and it is used a fuel for heaters, stoves, and tanks. An ideal fuel should be widely available, hydrogen rich, and potentially safe to transport and stock [163]. However, presence of high content of sulfurous compounds in JP-8 fuel poison the anodes of SOFCs and cylinder heads and exhaust valves in supercharged diesel engines. Lifetime of SOFCs can be enhanced with the use of JP-8 fuel with a concentration of <1ppmwS. Deep desulfurization of lighter fuels, such as gasoline, is usually carried out using hydrodesulfurization (HDS) [162,164]. However, several issues associated with HDS, including demanding operating conditions, requiring high temperatures and elevated pressures; non-selective hydrogenation; inefficiency for less reactive aromatic sulfur compounds (DMBT, TMBT); and low octane levels, call for new methods of desulfurization that are low cost and less energy intensive [165].

Mesoporous hierarchical MCM-41 or SBA-15 frameworks embedded with metallic active species and even metal oxides have been investigated in the past for adsorptive desulfurization of JP-5 aviation fuel. MCM-41 and SBA-15 were impregnated with Cu^+^ and Pd^2+^ ions by heating at 550 °C followed by spontaneous dispersion of monolayers. Under such synthetic conditions, the formed CuCl or PdCl_2_ coatings get homogenized with supporting frameworks [166]. The performance of Pd^2+^/MCM-41 was found to be better than Cu^+^/MCM-41 towards JP-5 as the breakthrough S capacity was 10.9 mg S/g for the former composite, while it was 7.7 mg S/g for the latter composite, and the saturation capacity of Pd^2+^ composite was 16.0 mg S/g and 14.4 mg S/g for the Cu^+^ composite. High desulfurization performance and better selectivity of Pd^2+^ composite can be attributed to its strong π interactions with the aromatic organosulfur compounds than Cu^+^. Interestingly, breakthrough capacity of Pd^2+^/SBA-15 (32.1 mg S/g) composites were much higher than the MCM-41 composites (10.9 mg S/g), even when the impregnation had 16% less Pd^2+^ in the former composites, and this higher performance can be correlated to the higher porosity and large pore size of SBA-15 compared to MCM-41 [166].

This work was followed by exploration of cuprous oxide impregnated mesoporous silica materials (MCM-41 and SBA-15) for ADS; however, the results contradicted with the previous work and claimed that large pore size and volume of SBA-15 was not beneficial [166]. Here, MCM-41 with a specific surface area of 523 m^2^/g outperformed as an adsorbent in comparison to SBA-15 with a lower surface area (400 m^2^/g) [167]. In these studies, a higher reduction temperature (700 °C) was perceived to be better in converting active species to Cu_2_O, leading to a greater adsorption capacity in case of MCM composite (12.8 mg S/g) compared to SBA-15 composite (9.6 mg S/g). The results also indicate that free transition metal ions exhibit higher performance in terms of saturation capacity and breakthrough capacity when compared to transition metal oxides. The saturation capacity of Cu^+^/SBA-15 was thrice that of Cu_2_O/SBA-15, and its breakthrough capacity was four times that of Cu_2_O/SBA-15. However, metal oxides could be regenerated more successfully in comparison to free metal ions due to their higher stability and strong bonding with surfaces via covalent interactions [167].

With the advent of studies on palladium catalysts, new efforts were exerted towards using precious metals ions like silver for desulfurization, and Ag^+^ impregnated mesoporous silicas were achieved through wet impregnation techniques [168]. In this case, the catalytic activity of Ag^+^/MCM-41 applied on JP-5 fuel was compared with Ag^+^/SBA-15, and breakthrough capacity of 15.7 mg S/g was achieved for the formed composite, and a value of 10.3 mg S/g was achieved for the latter. Saturation capacity, on the other hand, was 32.1 mg S/g for the MCM composite, whereas it was 29.2 mg S/g for the SBA composite. This data corroborates with the findings presented earlier by Wang et al., proving the high surface area of MCM-41 [166]. It was observed that Ag^+^/MCM-41 could be thermally recovered in air and could maintain 50% of its initial desulfurization efficiency, even after second and third cycles [168].

This work on silver-containing frameworks acted as a platform for further research on these materials and similar compounds have been evaluated for desulfurization of real, as well as model, fuels. Researchers have reported on the use of Ag-impregnated bulk MCM-41 for JP-5 fuel; however, there is still a huge demand for high edge adsorbents to be applied in advanced JP-8 fuel. A few years ago, Palomino et al. reported on using mesoporous silica nanoparticles as an extension to bulk MCM-41 for ADS of JP-8 aviation fuel with a sulfur concentration of 516 ppmw S [169]. This report was the first of its kind to compare commercial bulk MCM-41 with MCM-41 nanoparticles (MSNs) for JP-8 desulfurization. The prepared nanoparticles were spherical in shape and had an average diameter of 80 nm. Ag-impregnated composite catalytic materials were prepared using MCM-41 and MSN and their adsorption capacities were compared. Adsorption capacity of Ag–MCM-41 (24.5 mg S/g) was found to be lower than Ag–MSN (32.6 mg S/g) [169]. Maximum model fuel capacity was achieved when a silver loading of 18 wt% was used for Ag-MCM-41 and 20 wt% for Ag-MSN. Adsorption capacity and breakthrough S capacity of Ag-MSN (32.6 mg S/g, 0.98 mg S/g) were much higher than that of Ag-MCM-41(25.4 mg S/g, 0.21 mg S/g), owing to the high surface area of nanomaterials. Ag-MSN could also be recovered with diethyl ether and be reused with 70% efficiency [169]. To go in depth on other materials for jet fuels is beyond the scope of this review. For more details on catalytic materials for desulfurization of aviation fuels, we direct our readers to one excellent review provided by Tran et al. [165].

## 6. Summary

Key qualities that are widely present among ideal desulfurization materials include: (i) high porosity and large surface areas offering significant area and numerous active sites for adsorption within small volumes; (ii) catalytic materials and their precursors are economical; (iii) porous catalysts are usually embedded with active metal sites (in zerovalent, ionic, or oxide forms) for enriched adsorption; and (iv) finally, they are highly stable and can be recycled without significant loss of catalytic efficiency. These desulfurizing agents have unique surface and structural properties, including surface acidity, magnetic nature, photocatalytic activity, or the ability to homogenously disperse active sites and aiding the adsorption process.

Table 1 provides a summary of various functionalized mesoporous silica frameworks and their desulfurization performance. According to this table, it is clear that: (1) the sulfur adsorption capacity of MS frameworks impregnated with core-shell magnetic nanoparticles is significantly higher than that of MS frameworks embedded with photocatalytic nanoparticles; (2) although complete DBT conversion occurs within short period of time using subnano-MoO_3_/UMSN structure, LaW_10_/IL-SiO_2_ structure outperforms in terms of time and quantity for both small and large batches; (3) conversion performance of PW_11_@TMA-SBA-15 is remarkable in comparison to 0.05HPMo-IL/SBA-15, which shows lower desulfurization efficiency for various organosulfur compounds with higher reaction time; (4) the desulfurization performance of the hybrid periodic mesoporous silica (PW11@TMA-PMOE) is lower than that of the hybrid ordered mesoporous silica (PW11@TMA-SBA-15); and (5) a high conversion rate of an ideal catalyst should be accompanied by its high recyclability without significant loss in its efficiency.

## 7. Conclusions

Mesoporous silica frameworks and nanoparticles are the third generation of Si-based nanoporous materials that are promising for clean fuel applications. With increased research interest in the use of specialized MS frameworks for desulfurization of fuels, in this review, we discussed appropriate functionalization strategies vital to adsorb the sulfur rich molecules present in fuels and their oxidation to easily removable species. We examined how uniquely designed MS formulations are capable of encapsulating not only simple sulfur species but also bulky aromatic compounds, like 4,6-DMDBT. We compiled various templating technologies, as well as other methods, like molecular imprinted polymers, to generate catalytic MS frameworks and examined the diverse nature of how surface modification with ionic liquids, photocatalytic active species, magnetic core-shell nanoparticles, and carbonaceous active sites can significantly affect the outcome of sulfur adsorption and removal. Ultra-deep desulfurization of fuels, especially JP-8 type of aviation fuels for fuel cell applications, demand judicious methods of catalysis, where currently precious metal ions are used in the desulfurization process, and stability of porous frameworks is essential to make recovery and reuse of these materials viable. A thorough examination of not only the pore size but also the size of the bulk material, as well as their competitive and interfering interactions in the presence of other compounds in fuels, such as asphaltenes, remains to be explored and can provide insights on the performance of these particles and may provide valuable information related to the criteria of MS material design.

## Figures and Tables

**Table 1:** Desulfurization efficiency of different functionalized mesoporous silica materials.

Catalyst	Sulfur Species	Conversion % (time, min)	qm (mg-S per g-Cat)	Ref.
ZnO-Mesoporous TiO_2_-SiO_2_	DBT	97%	46.1	[78]
Mesoporous TiO_2_-SiO_2_-40	DBT	99%	13.7	[81]
Mo/10SiO_2_-550	4,6-DMDBT	100% (40 m)	-	[91]
WO_3_/SBA-15	4,6-DMDBT	100% (60 m)	-	[170]
15%MoO_3_/SBA-15	4,6-DMDBT	100% (60 m)	-	[171]
PW_11_@aptes-SBA-15	1-BT, DBT, 4-MDBT, 4,6-DMDBT	100% (60 m)	-	[100]
PW_11_@TMA-SBA-15	DBT	100% (30 m)	-	[101]
PW_11_@TMA-SBA-15	4-MDBT	100% (30 m)	-	[101]
PW_11_@TMA-SBA-15	4,6-DMDBT	100% (30 m)	-	[101]
PW_11_@TMA-SBA-15	1-BT	93.9% (30 m)	-	[101]
PW_11_@TMA-PMOE	DBT	98.2% (60 m)	-	[101]
PW_11_@TMA-PMOE	4-MDBT	99.0% (60 m)	-	[101]
PW_11_@TMA-PMOE	4,6-DMDBT	99.3% (60 m)	-	[101]
PW_11_@TMA-PMOE	1-BT	92.8% (60 m)	-	[101]
20%HPW/Zr-HMS	DBT	95.0% (30 m)	-	[59]
Mesoporous W-SiO_2_	BT, DBT, 4,6-MDBT	100% (60 m)	-	[24]
subnano-MoO_3_/UMSN	DBT	100% (15 m)	-	[106]
10% Mo/mSiO_2_ nanowire	DBT	100% (30 m)	-	[107]
W-mSiO_2_-450	DBT	99.6% (30 m)	-	[124]
[C_4_mim]_3_PW_12_O_40_@OMS	DBT	99.5% (60 m)	-	[125]
LaW_10_/IL-SiO_2_	DBT	100% (1 m, small batch)100% (25 m, large scale)	-	[128]
IL-3DOM SiO_2_	BT, DBT, 3-MBT, 4-MDBT, 4,6-DMDBT	100% (60 m)	-	[58]
PMo_12_@TBA-MSN	BT, DBT, 3-MBT, 4-MDBT, 4,6-DMDBT	98% (70 m)	-	[133]
PMo_12_@TMA-MSN		92% (70 m)	-	[133]
0.05HPMo-IL/SBA-15	DBT	90% (90 m)	-	[136]
0.05HPMo-IL/SBA-15	BT	71% (90 m)	-	[136]
0.05HPMo-IL/SBA-15	4,6-DMDBT	69.8% (90 m)	-	[136]
Fe_3_O_4_@mSiO_2_@DT-MIP	DBT	-	104.2	[141]
Fe_3_O_4_@mSiO_2_@DT-MIP	4-MDBT	-	113.6	[141]
Fe_3_O_4_@SiO_2_-IL/RS--MMS	4,6-DMDBT	98.3% (50 m)	-	[143]
Fe_3_O_4_@SiO_2_-IL/RS--MMS	4-MDBT	99.5% (50 m)	-	[143]
Fe_3_O_4_@SiO_2_-IL/RS--MMS	DBT	99.7% (50 m)	-	[143]

## References

[1] https://www.who.int/gho/publications/world_health_statistics/2018/en/.

[2] https://www.unece.org/info/about-unece/mission/unece-and-the-sdgs.html.

[3] https://ccacoalition.org/en/resources/global-strategy-introduce%C2%A0low-sulfur-fuels-and-cleaner-diesel-vehicles.

[4] Li H., Su S., Peng Y., Wu L., Xu K., Liu L., Qing M., Hu S., Wang Y., Xiang J. (2019). Effect of La-Modified Supporter on H_2_S Removal Performance of Mn/La/Al_2_O_3_ Sorbent in a Reducing Atmosphere. Ind. Eng. Chem. Res..

[5] Liu D., Zhou W., Wu J. (2016). CeO_2_–MnOx/ZSM-5 sorbents for H_2_S removal at high temperature. Chem. Eng. J..

[6] Guo L.F., Pan K.L., Lee H.M., Chang M.B. (2015). High-temperature gaseous H_2_S removal by Zn–Mn-based sorbent. Ind. Eng. Chem. Res..

[7] Wu M., Chang B., Lim T.-T., Oh W.-D., Lei J., Mi J. (2018). High-sulfur capacity and regenerable Zn-based sorbents derived from layered double hydroxide for hot coal gas desulfurization. J. Hazard. Mater..

[8] Zeng B., Yue H., Liu C., Huang T., Li J., Zhao B., Zhang M., Liang B. (2015). Desulfurization behavior of Fe–Mn-based regenerable sorbents for high-temperature H_2_S removal. Energy Fuels.

[9] Xia H., Liu B., Li Q., Huang Z., Cheung A.S.-C. (2017). High capacity Mn-Fe-Mo/FSM-16 sorbents in hot coal gas desulfurization and mechanism of elemental sulfur formation. Appl. Catal. B Environ..

[10] Chen C.-S., Budi C.S., Wu H.-C., Saikia D., Kao H.-M. (2017). Size-tunable Ni nanoparticles supported on surface-modified, cage-type mesoporous silica as highly active catalysts for CO_2_ hydrogenation. ACS Catal..

[11] Liang J., Liang Z., Zou R., Zhao Y. (2017). Heterogeneous catalysis in zeolites, mesoporous silica, and metal–organic frameworks. Adv. Mater..

[12] Manzano M., Vallet-Regí M. (2019). Mesoporous Silica Nanoparticles for Drug Delivery. Adv. Funct. Mater..

[13] Mendiratta S., Hussein M., Nasser H.A., Ali A.A.A. (2019). Multidisciplinary Role of Mesoporous Silica Nanoparticles in Brain Regeneration and Cancers: From Crossing the Blood–Brain Barrier to Treatment. Part. Part. Syst. Charact..

[14] Niu M., Yang H., Zhang X., Wang Y., Tang A. (2016). Amine-impregnated mesoporous silica nanotube as an emerging nanocomposite for CO_2_ capture. ACS Appl. Mater. Interfaces.

[15] Kankala R.K., Han Y.H., Na J., Lee C.H., Sun Z., Wang S.B., Kimura T., Ok Y.S., Yamauchi Y., Chen A.Z. (2020). Nanoarchitectured Structure and Surface Biofunctionality of Mesoporous Silica Nanoparticles. Adv. Mater..

[16] Shieh F.-K., Wang S.-C., Yen C.-I., Wu C.-C., Dutta S., Chou L.-Y., Morabito J.V., Hu P., Hsu M.-H., Wu K.C.-W. (2015). Imparting functionality to biocatalysts via embedding enzymes into nanoporous materials by a de novo approach: Size-selective sheltering of catalase in metal–organic framework microcrystals. J. Am.Chem. Soc..

[17] Sue Y.-C., Wu J.-W., Chung S.-E., Kang C.-H., Tung K.-L., Wu K.C.-W., Shieh F.-K. (2014). Synthesis of hierarchical micro/mesoporous structures via solid–aqueous interface growth: Zeolitic imidazolate framework-8 on siliceous mesocellular foams for enhanced pervaporation of water/ethanol mixtures. ACS Appl. Mater. Interfaces.

[18] Wang J., Xu Y., Ding B., Chang Z., Zhang X., Yamauchi Y., Wu K.C.W. (2018). Confined Self-Assembly in Two-Dimensional Interlayer Space: Monolayered Mesoporous Carbon Nanosheets with In-Plane Orderly Arranged Mesopores and a Highly Graphitized Framework. Angew. Chem. Int. Ed..

[19] Liao Y.-T., Matsagar B.M., Wu K.C.-W. (2018). Metal–organic framework (MOF)-derived effective solid catalysts for valorization of lignocellulosic biomass. ACS Sustain. Chem. Eng..

[20] Kaneti Y.V., Dutta S., Hossain M.S., Shiddiky M.J., Tung K.L., Shieh F.K., Tsung C.K., Wu K.C.W., Yamauchi Y. (2017). Strategies for improving the functionality of zeolitic imidazolate frameworks: Tailoring nanoarchitectures for functional applications. Adv. Mater..

[21] Klemm D., Kramer F., Moritz S., Lindström T., Ankerfors M., Gray D., Dorris A. (2011). Nanocelluloses: A New Family of Nature-Based Materials. Angew. Chem. Int. Ed..

[22] Lin N., Dufresne A. (2014). Nanocellulose in biomedicine: Current status and future prospect. Eur. Polym. J..

[23] Moon R.J., Martini A., Nairn J., Simonsen J., Youngblood J. (2011). Cellulose nanomaterials review: Structure, properties and nanocomposites. Chem. Soc. Rev..

[24] Shen D., Dai Y., Han J., Gan L., Liu J., Long M. (2018). A nanocellulose template strategy for the controllable synthesis of tungsten-containing mesoporous silica for ultra-deep oxidative desulfurization. Chem. Eng. J..

[25] Chen S.-Y., Cheng S. (2007). Acid-free synthesis of mesoporous silica using triblock copolymer as template with the aid of salt and alcohol. Chem. Mater..

[26] Zhao D., Huo Q., Feng J., Chmelka B.F., Stucky G.D. (1998). Nonionic triblock and star diblock copolymer and oligomeric surfactant syntheses of highly ordered, hydrothermally stable, mesoporous silica structures. J. Am.Chem. Soc..

[27] Chen D., Li Z., Yu C., Shi Y., Zhang Z., Tu B., Zhao D. (2005). Nonionic block copolymer and anionic mixed surfactants directed synthesis of highly ordered mesoporous silica with bicontinuous cubic structure. Chem. Mater..

[28] Kim T.-W., Kleitz F., Paul B., Ryoo R. (2005). MCM-48-like large mesoporous silicas with tailored pore structure: Facile synthesis domain in a ternary triblock copolymer− butanol− water system. J. Am. Chem. Soc..

[29] Flodström K., Alfredsson V., Källrot N. (2003). Formation of a New Ia3-d Cubic Meso-Structured Silica via Triblock Copolymer-Assisted Synthesis. J. Am. Chem. Soc..

[30] Yamada S., Motozuka S., Tagaya M. (2020). Synthesis of nanostructured silica/hydroxyapatite hybrid particles containing amphiphilic triblock copolymer for effectively controlling hydration layer structures with cytocompatibility. J. Mater. Chem. B.

[31] Sawant A., Raut D., Darvatkar N., Salunkhe M. (2011). Recent developments of task-specific ionic liquids in organic synthesis. Green Chem. Lett. Rev..

[32] Ward A.J., Pujari A.A., Costanzo L., Masters A.F., Maschmeyer T. (2011). Ionic liquid-templated preparation of mesoporous silica embedded with nanocrystalline sulfated zirconia. Nanoscale Res. Lett..

[33] Pujari A.A., Chadbourne J.J., Ward A.J., Costanzo L., Masters A.F., Maschmeyer T. (2009). The use of acidic task-specific ionic liquids in the formation of high surface area mesoporous silica. New J. Chem..

[34] Wang T., Kaper H., Antonietti M., Smarsly B. (2007). Templating behavior of a long-chain ionic liquid in the hydrothermal synthesis of mesoporous silica. Langmuir.

[35] Yamamoto E., Mori S., Shimojima A., Wada H., Kuroda K. (2017). Fabrication of colloidal crystals composed of pore-expanded mesoporous silica nanoparticles prepared by a controlled growth method. Nanoscale.

[36] Yang Z., Qi K., Rong J., Wang L., Liu Z., Yang Y. (2001). Template synthesis of 3-D bimodal ordered porous silica. Chin. Sci. Bull..

[37] Villaescusa L.A., Mihi A., Rodríguez I., García-Bennett A.E., Míguez H. (2005). Growth of mesoporous materials within colloidal crystal films by spin-coating. J. Phys. Chem. B.

[38] Peng W., Zhang Z., Rong M., Zhang M. (2019). Core-Shell Structure Design of Hollow Mesoporous Silica Nanospheres Based on Thermo-Sensitive PNIPAM and pH-Responsive Catechol-Fe^3+^ Complex. Polymers.

[39] Yi G.-R., Moon J.H., Yang S.-M. (2001). Ordered macroporous particles by colloidal templating. Chem. Mater..

[40] Qin L., Shi W., Liu W., Yang Y., Liu X., Xu B. (2016). Surface molecularly imprinted polymers grafted on ordered mesoporous carbon nanospheres for fuel desulfurization. RSC Adv..

[41] Wang Y., Yang Y., Xu L., Zhang J. (2011). Bisphenol A sensing based on surface molecularly imprinted, ordered mesoporous silica. Electrochim. Acta.

[42] Pan S.-D., Shen H.-Y., Zhou L.-X., Chen X.-H., Zhao Y.-G., Cai M.-Q., Jin M.-C. (2014). Controlled synthesis of pentachlorophenol-imprinted polymers on the surface of magnetic graphene oxide for highly selective adsorption. J. Mater. Chem. A.

[43] Zhang Z., Li J., Fu L., Liu D., Chen L. (2015). Magnetic molecularly imprinted microsensor for selective recognition and transport of fluorescent phycocyanin in seawater. J. Mater. Chem. A.

[44] Chandra Srivastava V. (2012). An evaluation of desulfurization technologies for sulfur removal from liquid fuels. RSC Adv..

[45] Austin G.T. (1984). Shreve’s Chemical Process Industries.

[46] Bej S.K., Maity S.K., Turaga U.T. (2004). Search for an efficient 4, 6-DMDBT hydrodesulfurization catalyst: A review of recent studies. Energy Fuels.

[47] Egorova M., Prins R. (2004). Competitive hydrodesulfurization of 4, 6-dimethyldibenzothiophene, hydrodenitrogenation of 2-methylpyridine, and hydrogenation of naphthalene over sulfided NiMo/γ-Al_2_O_3_. J. Catal..

[48] Li X., Wang A., Egorova M., Prins R. (2007). Kinetics of the HDS of 4, 6-dimethyldibenzothiophene and its hydrogenated intermediates over sulfided Mo and NiMo on γ-Al_2_O_3_. J. Catal..

[49] Layan Savithra G.H., Bowker R.H., Carrillo B.A., Bussell M.E., Brock S.L. (2013). Mesoporous matrix encapsulation for the synthesis of monodisperse Pd_5_P_2_ nanoparticle hydrodesulfurization catalysts. ACS Appl. Mater. Interfaces.

[50] Huirache-Acuña R., Nava R., Peza-Ledesma C.L., Lara-Romero J., Alonso-Núez G., Pawelec B., Rivera-Muñoz E.M. (2013). SBA-15 mesoporous silica as catalytic support for hydrodesulfurization catalysts. Materials.

[51] Marafi M., Furimsky E. (2017). Hydroprocessing catalysts containing noble metals: Deactivation, regeneration, metals reclamation, and environment and safety. Energy Fuels.

[52] Kumaran G.M., Garg S., Soni K., Kumar M., Sharma L., Rama Rao K., Dhar G.M. (2007). Effect of Al-SBA-15 support on catalytic functionalities of hydrotreating catalysts. II. Effect of variation of molybdenum and promoter contents on catalytic functionalities. Ind. Eng. Chem. Res..

[53] Soni K., Rana B., Sinha A., Bhaumik A., Nandi M., Kumar M., Dhar G. (2009). 3-D ordered mesoporous KIT-6 support for effective hydrodesulfurization catalysts. Appl. Catal. B Environ..

[54] Sorensen A.C., Fuller B.L., Eklund A.G., Landry C.C. (2004). Mo-Doped mesoporous silica for thiophene hydrodesulfurization: Comparison of materials and methods. Chem. Mater..

[55] Danforth S.J., Liyanage D.R., Hitihami-Mudiyanselage A., Ilic B., Brock S.L., Bussell M.E. (2016). Probing hydrodesulfurization over bimetallic phosphides using monodisperse Ni_2_-xM_x_P nanoparticles encapsulated in mesoporous silica. Surf. Sci..

[56] Chiranjeevi T., Kumar P., Maity S., Rana M., Dhar G.M., Rao T.P. (2001). Characterization and hydrodesulfurization catalysis on WS_2_ supported on mesoporous Al–HMS material. Microporous Mesoporous Mater..

[57] Campos-Martin J., Capel-Sanchez M., Fierro J. (2004). Highly efficient deep desulfurization of fuels by chemical oxidation. Green Chem..

[58] Chen X., Zhang M., Wei Y., Li H., Liu J., Zhang Q., Zhu W., Li H. (2018). Ionic liquid-supported 3DOM silica for efficient heterogeneous oxidative desulfurization. Inorg. Chem. Front..

[59] Yuzbashi S., Mousazadeh M., Ramezani N., Sid Kalal H., Sabour B. (2020). Mesoporous zirconium–silica nanocomposite modified with heteropoly tungstophosphoric acid catalyst for ultra-deep oxidative desulfurization. Appl. Organomet. Chem..

[60] Gao M., Zhang G., Tian M., Liu B., Chen W. (2019). Oxidative desulfurization of dibenzothiophene by central metal ions of chlorophthalocyanines-tetracarboxyl complexes. Inorg. Chim. Acta.

[61] Javadli R., De Klerk A. (2012). Desulfurization of heavy oil. Appl. Petrochem. Res..

[62] Zhu W., Li H., Jiang X., Yan Y., Lu J., He L., Xia J. (2008). Commercially available molybdic compound-catalyzed ultra-deep desulfurization of fuels in ionic liquids. Green Chem..

[63] Rajendran A., Cui T.-Y., Fan H.-X., Yang Z., Feng J., Li W. (2020). A comprehensive review on oxidative desulfurization catalysts targeting clean energy and environment. J. Mater. Chem. A.

[64] Hossain M.N., Park H.C., Choi H.S. (2019). A comprehensive review on catalytic oxidative desulfurization of liquid fuel oil. Catalysts.

[65] Houda S., Lancelot C., Blanchard P., Poinel L., Lamonier C. (2018). Oxidative desulfurization of heavy oils with high sulfur content: A review. Catalysts.

[66] Li J., Yang Z., Li S., Jin Q., Zhao J. (2020). Review on oxidative desulfurization of fuel by supported heteropolyacid catalysts. J. Ind. Eng. Chem..

[67] Ja’fari M., Ebrahimi S.L., Khosravi-Nikou M.R. (2018). Ultrasound-assisted oxidative desulfurization and denitrogenation of liquid hydrocarbon fuels: A critical review. Ultrason. Sonochem..

[68] Kirkwood K., Ebert S., Foght J., Fedorak P., Gray M. (2005). Bacterial biodegradation of aliphatic sulfides under aerobic carbon-or sulfur-limited growth conditions. J. Appl. Microbiol..

[69] Kirkwood K.M., Foght J.M., Gray M.R. (2007). Selectivity among organic sulfur compounds in one-and two-liquid-phase cultures of Rhodococcus sp. strain JVH1. Biodegradation.

[70] Kirkwood K.M., Andersson J.T., Fedorak P.M., Foght J.M., Gray M.R. (2007). Sulfur from benzothiophene and alkylbenzothiophenes supports growth of Rhodococcus sp. strain JVH1. Biodegradation.

[71] McFarland B.L., Boron D.J., Deever W., Meyer J., Johnson A.R., Atlas R.M. (1998). Biocatalytic sulfur removal from fuels: Applicability for producing low sulfur gasoline. Crit. Rev. Microbiol..

[72] Gupta N., Roychoudhury P., Deb J. (2005). Biotechnology of desulfurization of diesel: Prospects and challenges. Appl. Microbiol. Biotechnol..

[73] Sadare O.O., Obazu F., Daramola M.O. (2017). Biodesulfurization of petroleum distillates—Current status, opportunities and future challenges. Environments.

[74] Xu W., Li Y. (2015). Alkylation desulfurization of the C9 fraction over Amberlyst 36 resin. RSC Adv..

[75] Han D.Y., Chen C.J., Zhang F.J. (2011). Deep Desulfurization of Light Oil Using the Imitating Silver Salt ILs Method. Petrol. Sci. Technol..

[76] He J., Wu P., Wu Y., Li H., Jiang W., Xun S., Zhang M., Zhu W., Li H. (2017). Taming interfacial oxygen vacancies of amphiphilic tungsten oxide for enhanced catalysis in oxidative desulfurization. ACS Sustain. Chem. Eng..

[77] Wang X.-S., Huang Y.-B., Lin Z.-J., Cao R. (2014). Phosphotungstic acid encapsulated in the mesocages of amine-functionalized metal–organic frameworks for catalytic oxidative desulfurization. Dalton Trans..

[78] Zhou K., Ding Y., Zhang L., Wu H., Guo J. (2020). Synthesis of mesoporous ZnO/TiO _2_–SiO_2_ composite material and its application in photocatalytic adsorption desulfurization without the addition of an extra oxidant. Dalton Trans..

[79] Saravanan R., Karthikeyan S., Gupta V., Sekaran G., Narayanan V., Stephen A. (2013). Enhanced photocatalytic activity of ZnO/CuO nanocomposite for the degradation of textile dye on visible light illumination. Mater. Sci. Eng. C.

[80] Wang Z., Hu T., He H., Fu Y., Zhang X., Sun J., Xing L., Liu B., Zhang Y., Xue X. (2018). Enhanced H2 production of TiO_2_/ZnO nanowires Co-using solar and mechanical energy through piezo-photocatalytic effect. ACS Sustain. Chem. Eng..

[81] Qin B., Shen Y., Xu B., Zhu S., Li P., Liu Y. (2018). Mesoporous TiO_2_–SiO_2_ adsorbent for ultra-deep desulfurization of organic-S at room temperature and atmospheric pressure. RSC Adv..

[82] Meizhen X., Lina Y., Jian L. (2017). Photocatalytic oxidative desulfurization of dibenzothiophene on TiO_2_ modified bimodal mesoporous silica. China Pet. Process. Petrochem.Technol..

[83] Zaccariello G., Moretti E., Storaro L., Riello P., Canton P., Gombac V., Montini T., Rodriguez-Castellon E., Benedetti A. (2014). TiO_2_–mesoporous silica nanocomposites: Cooperative effect in the photocatalytic degradation of dyes and drugs. RSC Adv..

[84] Julião D., Gomes A.C., Pillinger M., Valença R., Ribeiro J.C., de Castro B., Gonçalves I.S., Cunha Silva L., Balula S.S. (2016). Zinc-Substituted Polyoxotungstate@ amino-MIL-101 (Al)–An Efficient Catalyst for the Sustainable Desulfurization of Model and Real Diesels. Eur. J. Inorg. Chem..

[85] Genovese M., Lian K. (2017). Polyoxometalate modified pine cone biochar carbon for supercapacitor electrodes. J. Mater. Chem. A.

[86] Ghahramaninezhad M., Soleimani B., Shahrak M.N. (2018). A simple and novel protocol for Li-trapping with a POM/MOF nano-composite as a new adsorbent for CO_2_ uptake. New J. Chem..

[87] Xun S., Zhu W., Chang Y., Li H., Zhang M., Jiang W., Zheng D., Qin Y., Li H. (2016). Synthesis of supported SiW_12_O_40_-based ionic liquid catalyst induced solvent-free oxidative deep-desulfurization of fuels. Chem. Eng. J..

[88] Li S.-W., Li J.-R., Gao Y., Liang L.-L., Zhang R.-L., Zhao J.-S. (2017). Metal modified heteropolyacid incorporated into porous materials for a highly oxidative desulfurization of DBT under molecular oxygen. Fuel.

[89] Shen D., Yang J., Li X., Zhou L., Zhang R., Li W., Chen L., Wang R., Zhang F., Zhao D. (2014). Biphase stratification approach to three-dimensional dendritic biodegradable mesoporous silica nanospheres. Nano Lett..

[90] Shen D., Chen L., Yang J., Zhang R., Wei Y., Li X., Li W., Sun Z., Zhu H., Abdullah A.M. (2015). Ultradispersed palladium nanoparticles in three-dimensional dendritic mesoporous silica nanospheres: Toward active and stable heterogeneous catalysts. ACS Appl. Mater. Interfaces.

[91] Zhang M., Liu J., Yang J., Chen X., Wang M., Li H., Zhu W., Li H. (2019). Molybdenum-containing dendritic mesoporous silica spheres for fast oxidative desulfurization in fuel. Inorg. Chem. Front..

[92] Casuscelli S., Crivello M., Perez C., Ghione G., Herrero E., Pizzio L., Vázquez P., Cáceres C., Blanco M. (2004). Effect of reaction conditions on limonene epoxidation with H_2_O_2_ catalyzed by supported Keggin heteropolycompounds. Appl. Catal. A Gen..

[93] Granadeiro C.M., Barbosa A.D., Silva P., Paz F.A.A., Saini V.K., Pires J., de Castro B., Balula S.S., Cunha-Silva L. (2013). Monovacant polyoxometalates incorporated into MIL-101 (Cr): Novel heterogeneous catalysts for liquid phase oxidation. Appl. Catal. A Gen..

[94] Ribeiro S., Barbosa A.D., Gomes A.C., Pillinger M., Gonçalves I.S., Cunha-Silva L., Balula S.S. (2013). Catalytic oxidative desulfurization systems based on Keggin phosphotungstate and metal-organic framework MIL-101. Fuel Process. Technol..

[95] Singh S., Patel A., Prakashan P. (2015). One pot oxidative esterification of aldehyde over recyclable cesium salt of nickel substituted phosphotungstate. Appl. Catal. A Gen..

[96] Coronel N.C., da Silva M.J. (2018). Lacunar keggin heteropolyacid salts: Soluble, solid and solid-supported catalysts. J. Clust. Sci..

[97] Abdalla Z.E.A., Li B. (2012). Preparation of MCM-41 supported (Bu_4_N)4H_3_(PW_11_O_39_) catalyst and its performance in oxidative desulfurization. Chem. Eng. J..

[98] Abdalla Z.E.A., Li B., Tufail A. (2009). Direct synthesis of mesoporous (C_19_H_42_N)4H_3_(PW_11_O_39_)/SiO_2_ and its catalytic performance in oxidative desulfurization. Colloids Surf. A Physicochem. Eng. Asp..

[99] Wu N., Li B., Liu Z., Han C. (2014). Synthesis of Keggin-type lacunary 11-tungstophosphates encapsulated into mesoporous silica pillared in clay interlayer galleries and their catalytic performance in oxidative desulfurization. Catal. Commun..

[100] Ribeiro S.O., Granadeiro C.M., Almeida P.L., Pires J., Capel-Sanchez M.C., Campos-Martin J.M., Gago S., De Castro B., Balula S.S. (2019). Oxidative desulfurization strategies using Keggin-type polyoxometalate catalysts: Biphasic versus solvent-free systems. Catal. Today.

[101] Ribeiro S.O., Granadeiro C.M., Corvo M.C., Pires J., Campos-Martin J.M., de Castro B., Balula S.S. (2019). Mesoporous Silica vs. Organosilica Composites to Desulfurize Diesel. Front. Chem..

[102] Ribeiro S.O., Almeida P., Pires J., de Castro B., Balula S.S. (2020). Polyoxometalate@ Periodic mesoporous organosilicas as active materials for oxidative desulfurization of diesels. Microporous Mesoporous Mater..

[103] Ni B., Wang X. (2016). Chemistry and properties at a sub-nanometer scale. Chem. Sci..

[104] Lu Y., Chen W. (2012). Sub-nanometre sized metal clusters: From synthetic challenges to the unique property discoveries. Chem. Soc. Rev..

[105] Wang J., Li X., Zhang S., Lu R. (2013). Facile synthesis of ultrasmall monodisperse “raisin–bun”-type MoO _3_/SiO_2_ nanocomposites with enhanced catalytic properties. Nanoscale.

[106] Wang J., Wu W., Ye H., Zhao Y., Wang W.-H., Bao M. (2017). MoO_3_ subnanoclusters on ultrasmall mesoporous silica nanoparticles: An efficient catalyst for oxidative desulfurization. RSC Adv..

[107] Dou J., Zeng H.C. (2014). Integrated Networks of Mesoporous Silica Nanowires and Their Bifunctional Catalysis–Sorption Application for Oxidative Desulfurization. ACS Catal..

[108] Ma Z., Yu J., Dai S. (2010). Preparation of inorganic materials using ionic liquids. Adv. Mater..

[109] Leng Y., Wang J., Zhu D., Ren X., Ge H., Shen L. (2009). Heteropolyanion-based ionic liquids: Reaction-induced self-separation catalysts for esterification. Angew. Chem. Int. Ed..

[110] Ma Y., Qing S., Wang L., Islam N., Guan S., Gao Z., Mamat X., Li H., Eli W., Wang T. (2015). Production of 5-hydroxymethylfurfural from fructose by a thermo-regulated and recyclable Brønsted acidic ionic liquid catalyst. RSC Adv..

[111] Jiang W., Zhu W., Chang Y., Chao Y., Yin S., Liu H., Zhu F., Li H. (2014). Ionic liquid extraction and catalytic oxidative desulfurization of fuels using dialkylpiperidinium tetrachloroferrates catalysts. Chem. Eng. J..

[112] Lü H., Wang S., Deng C., Ren W., Guo B. (2014). Oxidative desulfurization of model diesel via dual activation by a protic ionic liquid. J. Hazard. Mater..

[113] Ding Y., Zhang B., Gupta N., Su D.S. (2015). Heterogenization of homogenous reaction system on carbon surface with ionic liquid as mediator. Green Chem..

[114] Liu C., Zhang G., Zhao C., Li X., Li M., Na H. (2014). MOFs synthesized by the ionothermal method addressing the leaching problem of IL–polymer composite membranes. Chem. Commun..

[115] Wan H., Chen C., Wu Z., Que Y., Feng Y., Wang W., Wang L., Guan G., Liu X. (2015). Encapsulation of heteropolyanion-based ionic liquid within the metal–organic framework MIL-100 (Fe) for biodiesel production. ChemCatChem.

[116] Patil J.D., Patil S.A., Pore D.M. (2015). A polymer supported ascorbate functionalized task specific ionic liquid: An efficient reusable catalyst for 1, 3-dipolar cycloaddition. RSC Adv..

[117] Sharma P., Gupta M. (2015). Silica functionalized sulphonic acid coated with ionic liquid: An efficient and recyclable heterogeneous catalyst for the one-pot synthesis of 1, 4-dihydropyridines under solvent-free conditions. Green Chem..

[118] Sidhpuria K.B., Daniel-da-Silva A.L., Trindade T., Coutinho J.A. (2011). Supported ionic liquid silica nanoparticles (SILnPs) as an efficient and recyclable heterogeneous catalyst for the dehydration of fructose to 5-hydroxymethylfurfural. Green Chem..

[119] Zhang J., Wang A., Li X., Ma X. (2011). Oxidative desulfurization of dibenzothiophene and diesel over [Bmim] 3PMo12O40. J. Catal..

[120] Virtanen P., Mikkola J.-P., Toukoniitty E., Karhu H., Kordas K., Eränen K., Wärnå J., Salmi T. (2009). Supported ionic liquid catalysts—From batch to continuous operation in preparation of fine chemicals. Catal. Today.

[121] Bordoloi A., Sahoo S., Lefebvre F., Halligudi S. (2008). Heteropoly acid-based supported ionic liquid-phase catalyst for the selective oxidation of alcohols. J. Catal..

[122] Sadeghzadeh S.M. (2015). A heteropolyacid-based ionic liquid immobilized onto fibrous nano-silica as an efficient catalyst for the synthesis of cyclic carbonate from carbon dioxide and epoxides. Green Chem..

[123] Eguizábal A., Lemus J., Urbiztondo M., Moschovi A., Ntais S., Soler J., Pina M. (2011). Ammonium based ionic liquids immobilized in large pore zeolites: Encapsulation procedures and proton conduction performance. J. Power Sources.

[124] Gu Q., Zhu W., Xun S., Chang Y., Xiong J., Zhang M., Jiang W., Zhu F., Li H. (2014). Preparation of highly dispersed tungsten species within mesoporous silica by ionic liquid and their enhanced catalytic activity for oxidative desulfurization. Fuel.

[125] Zhang M., Li M., Chen Q., Zhu W., Li H., Yin S., Li Y., Li H. (2015). One-pot synthesis of ordered mesoporous silica encapsulated polyoxometalate-based ionic liquids induced efficient desulfurization of organosulfur in fuel. RSC Adv..

[126] Xu J., Zhao S., Chen W., Wang M., Song Y.F. (2012). Highly efficient extraction and oxidative desulfurization system using Na_7_H_2_LaW_10_O_36_⋅32H_2_O in [bmim] BF_4_ at room temperature. Chem. Eur. J..

[127] Xu J., Zhao S., Ji Y., Song Y.F. (2013). Deep Desulfurization by Amphiphilic Lanthanide-Containing Polyoxometalates in Ionic-Liquid Emulsion Systems under Mild Conditions. Chem. Eur. J..

[128] Chen Y., Song Y.F. (2014). Immobilization of LaW10 onto Ionic-Liquid-Modified Mesoporous Silica: Deep Desulfurization with Zero-Order Reaction Kinetics. ChemPlusChem.

[129] Wang Z., Fan X., Han D., Gu F. (2016). Structural and electronic engineering of 3DOM WO_3_ by alkali metal doping for improved NO_2_ sensing performance. Nanoscale.

[130] Wang J., Zhang W., Zheng Z., Gao Y., Ma K., Ye J., Yang Y. (2017). Enhanced thermal decomposition properties of ammonium perchlorate through addition of 3DOM core-shell Fe_2_O_3_/Co_3_O_4_ composite. J. Alloys Compd..

[131] Yue D., Lei J., Lina Z., Zhenran G., Du X., Li J. (2018). Oxidation desulfurization of fuels by using amphiphilic hierarchically meso/macroporous phosphotungstic acid/SiO_2_ catalysts. Catal. Letters.

[132] Du Y., Yang P., Zhou S., Li J., Du X., Lei J. (2018). Direct synthesis of ordered meso/macrostructured phosphotungstic acid/SiO_2_ by EISA method and its catalytic performance of fuel oil. Mater. Res. Bull..

[133] Mirante F., Gomes N., Branco L.C., Cunha-Silva L., Almeida P.L., Pillinger M., Gago S., Granadeiro C.M., Balula S.S. (2019). Mesoporous nanosilica-supported polyoxomolybdate as catalysts for sustainable desulfurization. Microporous Mesoporous Mater..

[134] Kozhevnikov I.V. (2002). Catalysts for fine chemical synthesis. Catalysis by Polyoxometalates.

[135] Okuhara T., Mizuno N., Misono M. (1996). Catalytic chemistry of heteropoly compounds. Advances in Catalysis.

[136] Xiong J., Zhu W., Ding W., Yang L., Zhang M., Jiang W., Zhao Z., Li H. (2015). Hydrophobic mesoporous silica-supported heteropolyacid induced by ionic liquid as a high efficiency catalyst for the oxidative desulfurization of fuel. RSC Adv..

[137] Huang W., Xu P., Yang W., Xu W. (2016). Thermosensitive molecularly imprinted polymers based on magnetic nanoparticles for the recognition of sulfamethazine. RSC Adv..

[138] Gao R., Mu X., Hao Y., Zhang L., Zhang J., Tang Y. (2014). Combination of surface imprinting and immobilized template techniques for preparation of core–shell molecularly imprinted polymers based on directly amino-modified Fe_3_O_4_ nanoparticles for specific recognition of bovine hemoglobin. J. Mater. Chem. B.

[139] Li Y., Li X., Chu J., Dong C., Qi J., Yuan Y. (2010). Synthesis of core-shell magnetic molecular imprinted polymer by the surface RAFT polymerization for the fast and selective removal of endocrine disrupting chemicals from aqueous solutions. Environ. Pollut..

[140] Men H.-F., Liu H.-Q., Zhang Z.-L., Huang J., Zhang J., Zhai Y.-Y., Li L. (2012). Synthesis, properties and application research of atrazine Fe_3_O_4_@ SiO_2_ magnetic molecularly imprinted polymer. Environ. Sci. Pollut. Res..

[141] Wang J., Wei J. (2017). Selective and simultaneous removal of dibenzothiophene and 4-methyldibenzothiophene using double-template molecularly imprinted polymers on the surface of magnetic mesoporous silica. J. Mater. Chem. A.

[142] Yue Q., Zhang Y., Jiang Y., Li J., Zhang H., Yu C., Elzatahry A.A., Alghamdi A., Deng Y., Zhao D. (2017). Nanoengineering of core–shell magnetic mesoporous microspheres with tunable surface roughness. J. Am.Chem. Soc..

[143] Jiang W., Jia H., Fan X., Dong L., Guo T., Zhu L., Zhu W., Li H. (2019). Ionic liquid immobilized on magnetic mesoporous microspheres with rough surface: Application as recyclable amphiphilic catalysts for oxidative desulfurization. Appl. Surf. Sci..

[144] Bian L., Li Y.-J., Li J., Nie J.-N., Dong F.-Q., Song M.-X., Wang L.-S., Dong H.-L., Li H.-L., Nie X.-Q. (2017). Photovoltage response of (XZn) Fe_2_O_4_-BiFeO_3_ (X= Mg, Mn or Ni) interfaces for highly selective Cr^3+^, Cd^2+^, Co^2+^ and Pb^2+^ ions detection. J. Hazard. Mater..

[145] Ko T.-H., Wang S., Chang F.-H., Chu C.-Y. (2017). Performance of ZnMn_2_O_4_/SiO_2_ sorbent for high temperature H_2_S removal from hot coal gas. RSC Adv..

[146] Liu Q., Zhang Z., Liu B., Xia H. (2018). Rare earth oxide doping and synthesis of spinel ZnMn_2_O_4_/KIT-1 with double gyroidal mesopores for desulfurization nature of hot coal gas. Appl. Catal. B Environ..

[147] Babaahamdi-Milani M., Nezamzadeh-Ejhieh A. (2016). A comprehensive study on photocatalytic activity of supported Ni/Pb sulfide and oxide systems onto natural zeolite nanoparticles. J. Hazard. Mater..

[148] Ejhieh A.N., Khorsandi M. (2010). Photodecolorization of Eriochrome Black T using NiS–P zeolite as a heterogeneous catalyst. J. Hazard. Mater..

[149] Sheikh-Mohseni M.H., Nezamzadeh-Ejhieh A. (2014). Modification of carbon paste electrode with Ni-clinoptilolite nanoparticles for electrocatalytic oxidation of methanol. Electrochim. Acta.

[150] Tamiji T., Nezamzadeh-Ejhieh A. (2018). A comprehensive study on the kinetic aspects and experimental design for the voltammetric response of a Sn (IV)-clinoptilolite carbon paste electrode towards Hg (II). J. Electroanal. Chem..

[151] Radko M., Rutkowska M., Kowalczyk A., Mikrut P., Święs A., Díaz U., Palomares A.E., Macyk W., Chmielarz L. (2020). Catalytic oxidation of organic sulfides by H_2_O_2_ in the presence of titanosilicate zeolites. Microporous Mesoporous Mater..

[152] Liu Q., Liu B., Liu Q., Xu R., Xia H. (2020). Lattice substitution and desulfurization kinetic analysis of Zn-based spinel sorbents loading onto porous silicoaluminophosphate zeolites. J. Hazard. Mater..

[153] Chao Y., Ju H., Luo J., Jin Y., Wang C., Xiong J., Wu P., Ji H., Zhu W. (2019). Synthesis of porous carbon via a waste tire leavening strategy for adsorptive desulfurization. RSC Adv..

[154] Shi Y., Zhang X., Wang L., Liu G. (2014). MOF-derived porous carbon for adsorptive desulfurization. AIChE J..

[155] Liu C., Yuan P., Duan A., Mei J., Zheng P., Meng Q., Cai A., Cheng T., Gong Y. (2019). Monodispersed dendritic mesoporous silica/carbon nanospheres with enhanced active site accessibility for selective adsorptive desulfurization. J. Mater. Sci..

[156] Ma X., Sun L., Song C. (2002). A new approach to deep desulfurization of gasoline, diesel fuel and jet fuel by selective adsorption for ultra-clean fuels and for fuel cell applications. Catal. Today.

[157] Wang L., Chen Y., Du L., Li S., Cai H., Liu W. (2013). Nickel-heteropolyacids supported on silica gel for ultra-deep desulfurization assisted by Ultrasound and Ultraviolet. Fuel.

[158] Zheng H., Sun Z., Chen X., Zhao Q., Wang X., Jiang Z. (2013). A micro reaction-controlled phase-transfer catalyst for oxidative desulfurization based on polyoxometalate modified silica. Appl. Catal. A Gen..

[159] Xun S., Zhu W., Zheng D., Zhang L., Liu H., Yin S., Zhang M., Li H. (2014). Synthesis of metal-based ionic liquid supported catalyst and its application in catalytic oxidative desulfurization of fuels. Fuel.

[160] Zhao Y., Wang J., Jiang H., Hu Y. (2015). Desulfurization performance of ether-functionalized imidazolium-based ionic liquids supported on porous silica gel. Energy Fuels.

[161] Safa M., Mokhtarani B., Mortaheb H.R., Tabar Heidar K., Sharifi A., Mirzaei M. (2017). Oxidative desulfurization of diesel fuel using a Brønsted acidic ionic liquid supported on silica gel. Energy Fuels.

[162] Samokhvalov A., Tatarchuk B.J. (2010). Review of experimental characterization of active sites and determination of molecular mechanisms of adsorption, desorption and regeneration of the deep and ultradeep desulfurization sorbents for liquid fuels. Catal. Rev..

[163] Hernández-Maldonado A.J., Yang R.T. (2004). Desulfurization of transportation fuels by adsorption. Catal. Rev..

[164] Yang R.T., Hernández-Maldonado A.J., Yang F.H. (2003). Desulfurization of transportation fuels with zeolites under ambient conditions. Science.

[165] Tran D.T., Palomino J.M., Oliver S.R. (2018). Desulfurization of JP-8 jet fuel: Challenges and adsorptive materials. RSC Adv..

[166] Wang Y., Yang R.T., Heinzel J.M. (2008). Desulfurization of jet fuel by π-complexation adsorption with metal halides supported on MCM-41 and SBA-15 mesoporous materials. Chem. Eng. Sci..

[167] Wang Y., Yang R.T., Heinzel J.M. (2009). Desulfurization of jet fuel JP-5 light fraction by MCM-41 and SBA-15 supported cuprous oxide for fuel cell applications. Ind. Eng. Chem. Res..

[168] Chen H., Wang Y., Yang F.H., Yang R.T. (2009). Desulfurization of high-sulfur jet fuel by mesoporous π-complexation adsorbents. Chem. Eng. Sci..

[169] Palomino J.M., Tran D.T., Hauser J.L., Dong H., Oliver S.R. (2014). Mesoporous silica nanoparticles for high capacity adsorptive desulfurization. J. Mater. Chem. A.

[170] Gonzalez J., Wang J., Chen L., Manriquez M., Dominguez J. (2017). Structural defects, Lewis acidity, and catalysis properties of mesostructured WO_3_/SBA-15 nanocatalysts. J. Phys. Chem. C.

[171] González J., Wang J.A., Chen L., Manríquez M., Salmones J., Limas R., Arellano U. (2018). Quantitative determination of oxygen defects, surface lewis acidity, and catalytic properties of mesoporous MoO_3_/SBA-15 catalysts. J. Solid State Chem..

